# A generalized density-modulated twist-splay-bend phase of banana-shaped particles

**DOI:** 10.1038/s41467-021-22413-8

**Published:** 2021-04-12

**Authors:** Massimiliano Chiappini, Marjolein Dijkstra

**Affiliations:** Soft Condensed Matter, Debye Institute for Nanomaterials Science, Department of Physics, Utrecht University, Utrecht, The Netherlands

**Keywords:** Liquid crystals, Statistical physics

## Abstract

In 1976, Meyer predicted that bend distortions of the nematic director field are complemented by deformations of either twist or splay, yielding twist-bend and splay-bend nematic phases, respectively. Four decades later, the existence of the splay-bend nematic phase remains dubious, and the origin of these spontaneous distortions uncertain. Here, we conjecture that bend deformations of the nematic director can be complemented by simultaneous distortions of both twist and splay, yielding a twist-splay-bend nematic phase. Using theory and simulations, we show that the coupling between polar order and bend deformations drives the formation of modulated phases in systems of curved rods. We find that twist-bend phases transition to splay-bend phases via intermediate twist-splay-bend phases, and that splay distortions are always accompanied by periodic density modulations due to the coupling of the particle curvature with the non-uniform curvature of the splayed director field, implying that the twist-splay-bend and splay-bend phases of banana-shaped particles are actually smectic phases.

## Abstract (web summary)

The so-called twist-bend and splay-bend nematic liquid crystal phases are important concepts for studying bent-core mesogens. Chiappini et al. use a theory/simulation approach to suggest that the transition proceed via a twist-splay-bend phase which may be obscured by density modulations.

## Introduction

The simplest and most common liquid crystal phase is the nematic phase, which is also the most relevant one for optoelectronic applications. The uniaxial nematic (N) phase consists of anisotropic particles that lack positional order but display orientational order as the particles are preferentially aligned along a so-called nematic director $\hat{{\bf{n}}}$. More exotic states of matter can be conjectured if the nematic director is allowed to vary in space, i.e. the average orientation of a particle at position **r** is defined by a nematic director field $\hat{{\bf{n}}}({\bf{r}})$. A well-known example is the chiral nematic or cholesteric (N^*^) phase, which is characterised by a nematic director that rotates as a helix around a chiral director with a cholesteric pitch length *p*, denoting the length scale associated to the helical periodicity.

**Fig. 1:**
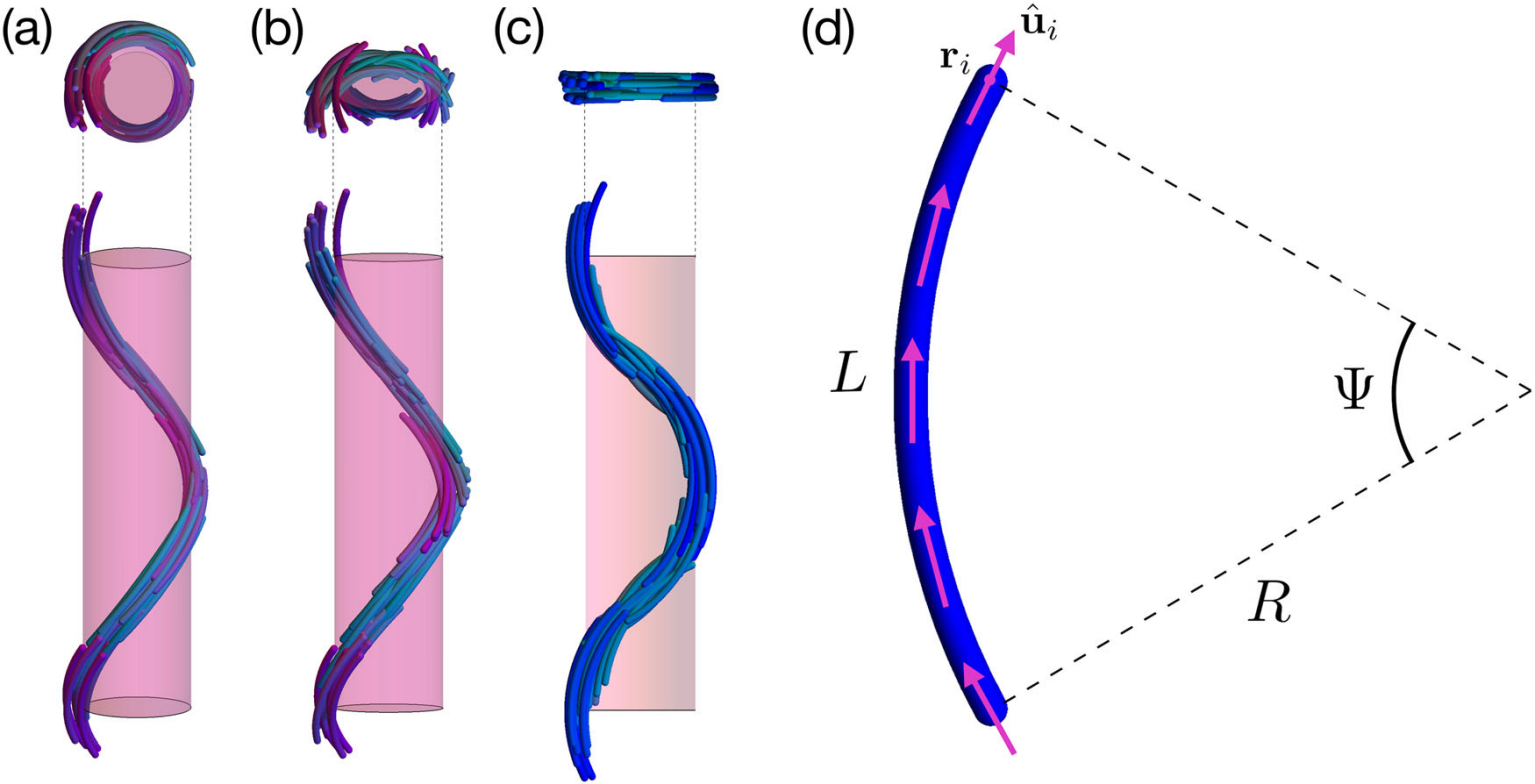
Schematics of a twist-bend, twist-splay-bend, and splay-bend nematic phase of curved rods. **a**–**c** Side and top views of the spatial modulations of the particle orientations in (**a**) a chiral twist-bend nematic (N_TB_) phase, **b** a chiral twist-splay-bend nematic (N_TSB_) phase and (**c**) an achiral splay-bend nematic (N_SB_) phase. **d** A hard curved spherocylinder consisting of a cylinder of length *L* and diameter *D* capped at both ends with a hemisphere of diameter *D* and bent with a radius of curvature *R* corresponding to an opening angle Ψ = *L*/*R*. In our generalized Maier–Saupe theory, this particle is modelled as a rigid chain of *M* segments with center-of-mass positions **r**_*i*_ and orientations ${\hat{{\bf{u}}}}_{i}$ tangent to the particle profile for *i* ∈ 1, ⋯ , *M*, sketched by the (pink) arrows pointing upwards along the arc.

An even more fascinating example is the twist-bend nematic (N_TB_) phase, recently discovered in experiments on bent-core mesogens^1–12^. The N_TB_ phase was already predicted by Meyer in 1976^13^ and by Dozov in 2001^14^ for banana-shaped particles that favor spontaneous bend deformations in the nematic director field. As a pure bend deformation cannot uniformly fill the three-dimensional space, local bend deformations have to be accompanied by either a spontaneous twist, yielding an N_TB_ phase, or by splay distortions, resulting into an oscillating splay-bend nematic (N_SB_) phase, see Fig. 1a, c. The N_TB_ phase is characterised by a nematic director field that precesses around a right circular cone with a pitch *p* and a conical angle 0 < *θ*_0_ < *π*/2. Hence, the N_TB_ phase is a chiral phase with local polar order and a uniform bend deformation. Because of the achirality of bent-core mesogens, the precession of the nematic director of the N_TB_ phase can be left- or right-handed. On the other hand, the nematic director field of the N_SB_ phase precesses over a flat isosceles triangle with maximum angle *θ*_0_, thereby preserving the achiral symmetry and oscillating between non-uniform bend and splay domains, see Supplementary Note 2.

Many fundamental questions regarding the N_TB_ phase are still open despite numerous theoretical and experimental investigations. Even the most basic question regarding the origin of bend deformations and the appearance of chiral symmetry in systems of achiral bent-shaped particles is still unresolved. While Meyer invoked that bend deformations originate from the spontaneous polar ordering of the particles due to either the molecular shape or the electrostatic polarization^13,15^, Dozov ignored the possibility of spontaneous polar order and explained the bend distortions by a negative bend elastic constant^14^. In addition, the relationship between the structure of the N_TB_ phase and the details of the constituent molecules is still not well understood. It is found experimentally that the observation of the N_TB_ phase depends sensitively on the molecular details.

Flexible bent-core molecules linked with an odd number of hydrocarbon atoms display N_TB_ phases but not the ones with an even-numbered linkage^16^. On the other hand, computer simulations demonstrate the existence of N_TB_ phases for both rigid and flexible banana-shaped molecules^17–20^. Flexibility also plays an important role in the stabilisation of N_TB_ phases as most rigid bent-core molecules form smectic (Sm) phases instead of nematic phases. Finally, the prediction of the surprisingly short pitch length and of the non-trivial tilt angle of the helicoidal nematic director field on the basis of the microscopic details of the particles is also of urgent interest for the design of optoelectronic materials.

**Fig. 2:**
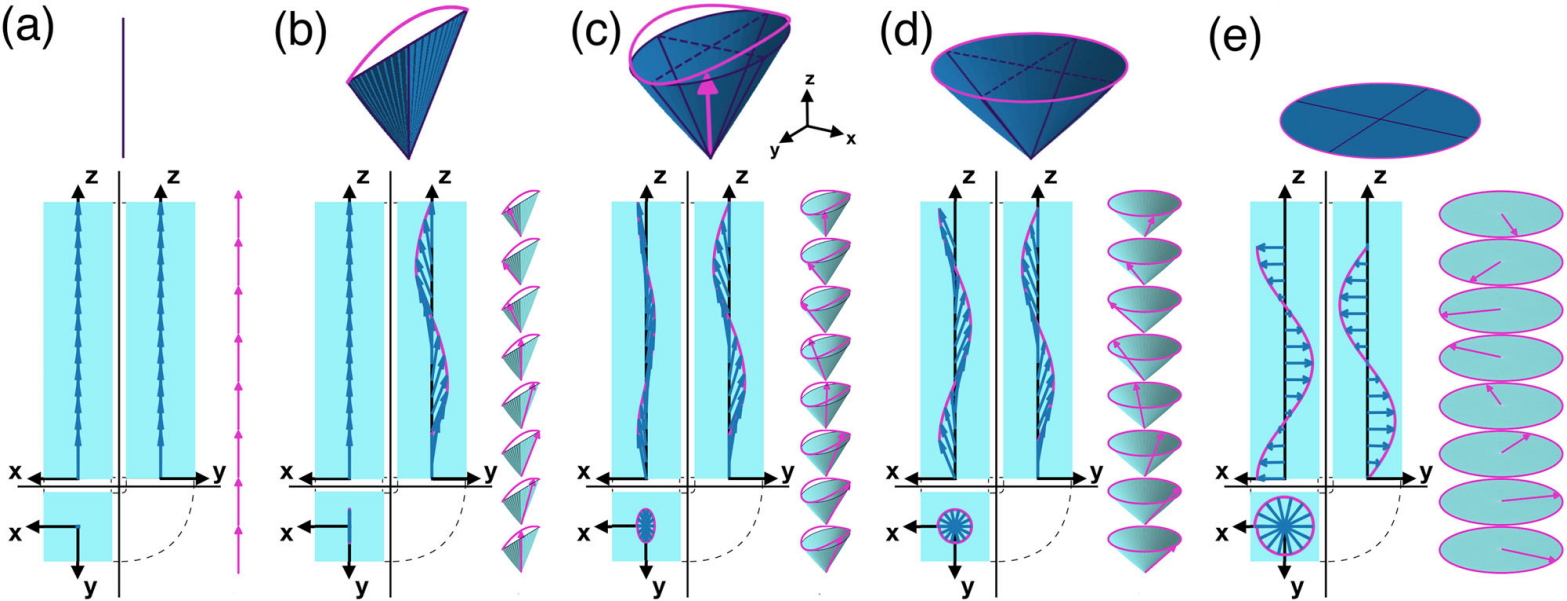
Nematic director field of a twist-splay-bend nematic phase. Precession cone (top panel) and orthogonal projection of the nematic director field ${\hat{{\bf{n}}}}_{{\rm{TSB}}}(z| {\theta }_{{\rm{a}}},{\theta }_{{\rm{b}}},q)$ (bottom panel) of several limiting cases of the twist-splay-bend nematic (N_TSB_) phase: **a** the uniaxial nematic (N) phase with *θ*_a_ = *θ*_b_ = 0, **b** the splay-bend nematic (N_SB_) phase with *θ*_b_ = 0, **c** the N_TSB_ phase with *θ*_a_ ≠ *θ*_b_, **d** the twist-bend (N_TB_) phase with *θ*_a_ = *θ*_b_, and (**e**) the cholesteric (N*) phase with *θ*_a_ = *θ*_b_ = *π*/2.

On the other hand, even though the Oseen–Frank theory predicts the N_SB_ to be more stable than the N_TB_ phase when the splay elastic constant is smaller than twice the twist elastic constant, experimental evidence of a N_SB_ phase is still lacking. Recent simulations suggest that an N_SB_ phase could be stabilised in lyotropic systems of banana-shaped particles^20^ but the presence of long-ranged density modulations questions the nematic nature of this N_SB_ phase. We also note that a transition from an N_TB_ phase to an unidentified density-modulated (Sm_X_) phase was recently observed in experiments^21^. Yet it is unclear what the physical mechanism is behind the N_SB_ phase and how the system transforms into an N_SB_ phase. Does the transformation from the N or N_TB_ phase proceed via a first-order or second-order phase transition? Here we conjecture that the transition from the N_TB_ to the N_SB_ phase may proceed via an intermediate phase that displays spatial modulations of both twist, splay, and bend, thereby extending Meyer’s speculations of 1976 by conjecturing that spontaneous bend deformations of the nematic director field can be accompanied by simultaneous deformations of both twist and splay. In this picture, twist deformations in the N_TB_ phase are gradually replaced by splay deformations, eventually resulting into an N_SB_ phase with pure splay and bend deformations. A similar scenario was recently discovered by studying the response of an N_TB_ phase to an external field^22,23^, which undergoes a structural change via an elliptical analogue of the N_TB_ phase to an N_SB_ phase upon increasing the field strength. This intermediate phase that we term the twist-splay-bend nematic (N_TSB_) phase is characterized by a nematic director that precesses around an elliptical cone with conical angles *θ*_a_ and *θ*_b_, see Fig. 2.

To shed light on the microscopic origin of the spatially modulated nematic phases and to better understand the transformation from the N_TB_ to an N_SB_ phase via a possible N_TSB_ phase, we develop a Maier–Saupe-like mean-field theory that takes into account not only the particle shape and interactions, but also the spatial modulations of the nematic director and density field in a variational fashion. We map out a theoretical phase diagram of curved spherocylinders that displays stable N_TB_, twist-splay-bend, and splay-bend phases, and test the predictions against simulations. We show that the twist-splay-bend and splay-bend (smectic) phases present periodic density modulations due to non-uniform deformations in the director field. Finally, we derive a relation between the particle curvature and the structure of the N_TB_ phase.

## Results

### A variational mean-field theory for spatially modulated liquid crystal phases

We generalize a recent Maier–Saupe theory for thermotropic bent-core mesogens^24^ to determine the phase behavior of curved spherocylinders with diameter *D*, length *L*, and radius of curvature *R* corresponding to a central angle Ψ = *L*/*R* (Fig. 1d). We describe a curved spherocylinder with centre-of-mass position **R** = (*X*, *Y*, *Z*) and orientation Ω = (*α*, *β*, *γ*) as a rigid chain of *M* segments of length *L*/*M*. We find that *M* = 10 segments is sufficient to account for the particle shape for the range of Ψ that we considered. Each segment *i*, with centre-of-mass position **r**_*i*_ and orientation ${\hat{{\bf{u}}}}_{i}$, is assumed to align preferentially along the local nematic director $\hat{{\bf{n}}}({{\bf{r}}}_{i})$ via an effective mean-field potential *βU*(**R**, Ω). In addition, we employ McMillan’s extension^25^ to account for possible density modulations along the (global) nematic director. The resulting effective mean-field potential reads

1 $$\beta U({\bf{R}},{{\Omega }})=-\frac{\beta \epsilon }{M}\left[S+\alpha \tau \cos \left(\frac{2\pi Z}{\lambda }\right)\right]\times \left(\mathop{\sum }\limits_{i = 1}^{M}{P}_{2}({\hat{{\bf{u}}}}_{i}\cdot \hat{{\bf{n}}}({{\bf{r}}}_{i}))\right),$$

where, *βϵ* is a dimensionless constant that quantifies the alignment strength, *P*_2_(*x*) = (3*x*^2^ − 1)/2 is the second-order Legendre polynomial, *β* = 1/*k*_B_*T* is the inverse temperature with *k*_B_ Boltzmann’s constant, *λ* is the periodicity of the density modulations, and *α* is a tunable parameter determining the tendency of the system to form density modulations. Furthermore, *S* and *τ* are the local nematic and the smectic order parameters, respectively,

2 $$S= 	\, \left\langle \frac{1}{M}\mathop{\sum }\limits_{i = 1}^{M}{P}_{2}({\hat{{\bf{u}}}}_{i}\cdot \hat{{\bf{n}}}({{\bf{r}}}_{i}))\right\rangle ,\; {\text{and}} \\ \tau =	 \, \left\langle \cos \left(\frac{2\pi Z}{\lambda }\right)\left(\frac{1}{M}\mathop{\sum }\limits_{i = 1}^{M}{P}_{2}({\hat{{\bf{u}}}}_{i}\cdot \hat{{\bf{n}}}({{\bf{r}}}_{i}))\right)\right\rangle ,$$

where 〈 ⋯ 〉 = ∫d**R**dΩ*f*(**R**, Ω) ⋯ denotes the ensemble average with the probability distribution function $f({\bf{R}},{{\Omega }})=\exp [-\beta U({\bf{R}},{{\Omega }})]/Q$ for the position and orientation of a curved spherocylinder, and $Q=\int \,\text{d}\,{\bf{R}}\,\text{d}\,{{\Omega }}\ \exp [-\beta U({\bf{R}},{{\Omega }})]$ is the partition function.

As a variational ansatz for $\hat{{\bf{n}}}({\bf{r}})$ we employ the nematic director field ${\hat{{\bf{n}}}}_{{\rm{TSB}}}(z| {\theta }_{{\rm{a}}},{\theta }_{{\rm{b}}},q)$ of a generic N_TSB_ phase with conical angle *θ*_a_ and *θ*_b_, wavenumber *q*, and the helical axis along the *z* − direction

3 $${\hat{{\bf{n}}}}_{{\rm{TSB}}}(z| {\theta }_{{\rm{a}}},{\theta }_{{\rm{b}}},q)= 	\,\sin ({\theta }_{{\rm{b}}})\cos (qz)\,{\bf{i}}+\sin ({\theta }_{{\rm{a}}})\sin (qz)\,{\bf{j}}\\ 	\,+\sqrt{1-{\sin }^{2}({\theta }_{{\rm{b}}}){\cos }^{2}(qz)-{\sin }^{2}({\theta }_{{\rm{a}}}){\sin }^{2}(qz)}\;{\bf{k}}.$$

We note that the N_TSB_ phase reduces to an N_TB_ phase with a circular precession cone when *θ*_a_ = *θ*_b_ = *θ*_0_, whereas for *θ*_a_ = *θ*_b_ = *π*/2 the circular cone reduces to a flat circle resulting into an N^*^ phase as the precession of the nematic director reduces to a simple twist around the phase director. If either *θ*_a_ or *θ*_b_ vanishes, the elliptical cone collapses onto a flat isosceles triangle and an N_SB_ phase is obtained with nematic director field ${\hat{{\bf{n}}}}_{{\rm{TSB}}}(z| {\theta }_{0},0,q)=\sin ({\theta }_{0})\sin (qz)\,{\bf{j}}+\sqrt{1-{\sin }^{2}({\theta }_{0}){\sin }^{2}(qz)}\, {\bf{k}}$ which differs from the expression ${\hat{{\bf{n}}}}_{{\rm{SB}}}(z)=\sin ({\theta }_{0}\sin (qz))\, {\bf{j}}+\cos ({\theta }_{0}\sin (qz))\,{\bf{k}}$ given by Dozov^14^. However, for small conical angles the two expressions for the nematic director fields are indistinguishable, see Supplementary Note 1. Finally, for *θ*_a_ = *θ*_b_ = 0 the cone becomes a simple line, and the N_TSB_ simply reduces to a uniaxial N phase. Hence, all the above-mentioned nematic phases are limiting cases of the N_TSB_ phase, see Fig. 2. In the following, we distinguish N_TB_, N_TSB_, and N_SB_ states based on their ellipticity *e* = *θ*_b_/*θ*_a_: we label N_TB_ the states with *e* > 0.8, N_TSB_ the states with 0.2 < *e* < 0.8, and N_SB_ the states with *e* < 0.2. We note that the thresholds *e* = 0.2 and *e* = 0.8 — necessary because of the statistical error on *e* — are arbitrary, and every state with ellipticity *e* ≠ 0, 1 is, in principle, an N_TSB_ state.

As *βU*(**R**, Ω) only depends on the *Z*-component of **R** with period *p*, we restrict all integrations over **R** to integrations over *Z* ∈ [0, *p*]. The onset of orientational and/or positional order corresponds to a change of entropy per particle ${{\Delta }}{\mathcal{S}}/N=-{k}_{{\rm{B}}}\int \,{\text{d}}Z{\text{d}}\,{{\Omega }}\ f(Z,{{\Omega }}) {\mathrm{log}}\,[16{\pi }^{3}f(Z,{{\Omega }})/q]=\langle U(Z,{{\Omega }})\rangle /T-{k}_{{\rm{B}}}\mathrm{log}\,\left(16{\pi }^{3}/qQ\right)$and to a change of energy per particle Δ*U*/*N* = 〈*U*(*Z*, Ω)〉/2. Using Eq. (2), we obtain the change of free energy per particle relative to the isotropic state

4 $$\frac{\beta {{\Delta }}F}{N}=\frac{\beta {{\Delta }}U}{N}-\frac{{{\Delta }}{\mathcal{S}}}{{k}_{{\rm{B}}}N}=\beta \epsilon \left(\frac{{S}^{2}+\alpha {\tau }^{2}}{2}\right)-\mathrm{log}\,\left(\frac{qQ}{16{\pi }^{3}}\right).$$

Minimizing Δ*F* with respect to *S*, *τ*, *λ*, and the variational parameters *θ*_a_, *θ*_b_, and *q* of the nematic director field ${\hat{{\bf{n}}}}_{{\rm{TSB}}}(z| {\theta }_{{\rm{a}}},{\theta }_{{\rm{b}}},q)$ yields the equilibrium phase at temperature *k*_B_*T*/*ϵ*. We note that since the nematic director ${\hat{{\bf{n}}}}_{{\rm{TSB}}}(z| {\theta }_{{\rm{a}}},{\theta }_{{\rm{b}}},q)$ already imposes a periodicity with pitch length *p* = 2*π*/*q* in the system, *p* must be a multiple of the periodicity *λ* of the density modulations, *i.e*.*p* = *nλ* with $n\in {\mathbb{N}}$. As the numerical minimization of Δ*F* always yields non-integer values of *n* close to 2 in spatially modulated phases, we impose *n* = 2 in all considered cases.

**Fig. 3:**
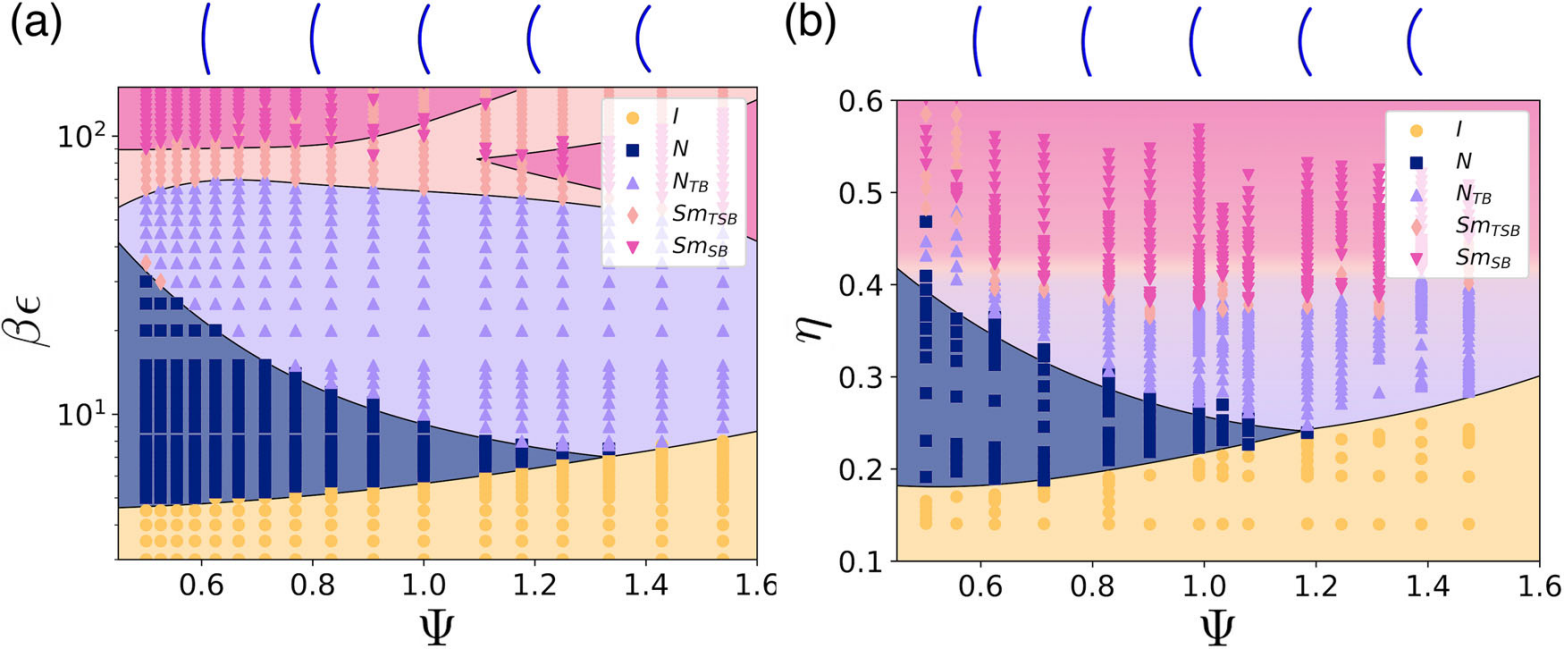
Phase diagram of curved spherocylinders. Phase behaviour of hard curved spherocylinders as (**a**) predicted by theory, as a function of inverse temperature *βϵ* and particle curvature Ψ, and (**b**) obtained from simulations, as a function of packing fraction *η* and Ψ. Both phase diagrams exhibit an isotropic (I) phase at low *βϵ* and *η*, respectively. For small particle curvatures Ψ the I phase transitions into an uniaxial nematic (N) phase upon increasing *βϵ* or *η*. The stability range of the N phase decreases with Ψ and disappears at Ψ ≳ 1.2. Upon increasing *βϵ* or *η* further, the twist-bend nematic (N_TB_) phase transforms into a twist-splay-bend smectic (Sm_TSB_) phase, and eventually into a splay-bend smectic (Sm_SB_) phase. The splay deformations in the nematic director field are accompanied by density modulations. Source data are provided as a Source Data file.

In Fig. 3a we show the resulting phase diagram from Maier–Saupe theory for a system of curved spherocylinders as a function of Ψ and inverse temperature *βϵ*, where we set *α* = 0.05. At low curvature, *i.e*. small Ψ, the I phase transforms into a uniaxial N phase and subsequently into an N_TB_ phase upon increasing *βϵ*. However, the stability range of the N phase shrinks with increasing particle curvature, eventually disappearing at Ψ ≳ 1.2. Our results show that deformations of the nematic director field become more pronounced with increasing particle curvature Ψ until the I-N phase transition is replaced by a direct I-N_TB_ transition^20,24,26^.

Moreover, our generalized Maier–Saupe theory predicts that upon increasing *βϵ* further, the nematic director field exhibits not only twist and bend modulations but also splay deformations, resulting in a twist-splay-bend phase. Upon lowering the temperature further, the twist deformations are gradually replaced by splay deformations, eventually yielding a splay-bend phase at sufficiently high *βϵ*. In particular, we find two distinct regions of splay-bend phases, one at low particle curvature and one at high curvature, the latter transforming into a re-entrant twist-splay-bend phase with increasing *βϵ*. Intriguingly, our theory predicts that the onset of splay deformations is accompanied by density modulations. We therefore refer to these phases as twist-splay-bend smectic (Sm_TSB_) and splay-bend smectic (Sm_SB_) phases rather than N_TSB_ and N_SB_ phases. This finding is also supported by the observation that in the case of *α* = 0, i.e. without McMillan’s extension to describe density modulations, the phase diagram displays only stable I, N, and N_TB_ phases. The N_SB_ phase is thus unstable in the case of a homogeneous density field. Furthermore, we find that varying the value of *α* > 0 results only in a temperature shift of the transition from N_TB_ to the Sm_TSB_ and Sm_SB_ phase, whereas the amplitude of the density modulations remains unaffected. This confirms that splay deformations of the nematic director field are inherently associated with the appearance of density modulations, as already speculated by De Gennes and Meyer, four decades ago^27,28^.

### Monte Carlo simulations of hard curved spherocylinders in bulk and sedimentation

**Fig. 4:**
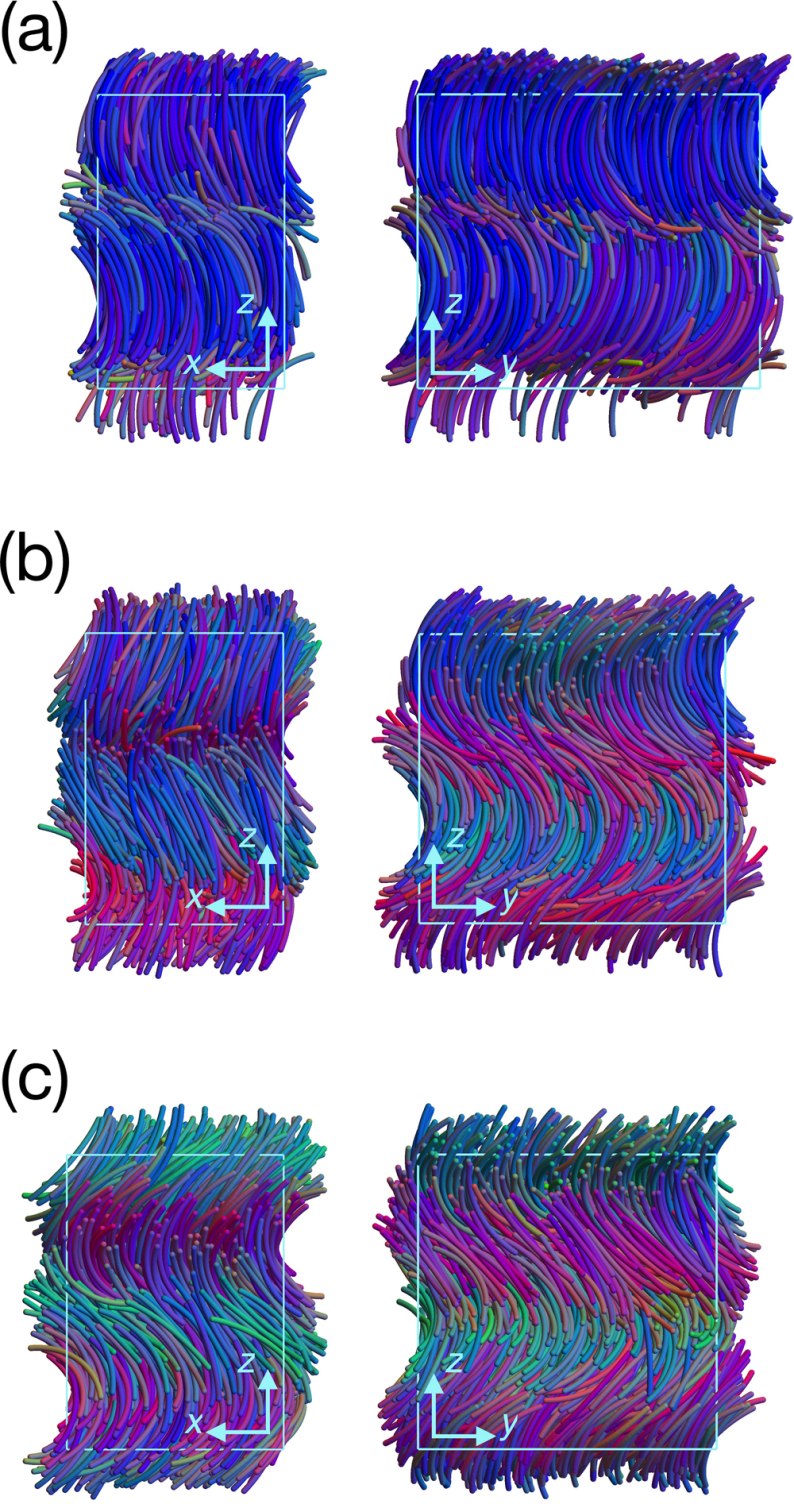
Splay-bend smectic Sm_SB_, twist-splay-bend smectic Sm_TSB_, and twist-bend nematic N_TB_ phases. Typical configurations of (**a**) an Sm_SB_ phase with conical angles *θ*_a_ ~ 0.76 and *θ*_b_ ~ 0 at packing fraction *η* = 0.394 (box size 55.5*D* × 30.3*D* × 47.7*D*), **b** an Sm_TSB_ phase with conical angles *θ*_a_ ~ 0.87 and *θ*_b_ ~ 0.51 at packing fraction *η* = 0.371 (box size 52.0*D* × 33.4*D* × 49.1*D*), and (**c**) an N_TB_ phase with conical angles *θ*_a_ ~ *θ*_b_ ~ 0.83 at packing fraction *η* = 0.354 (box size 49.9*D*. 3*D* × 49.3*D*) along an expansion of a system of hard curved spherocylinders of length *L*/*D* = 19 and opening angle Ψ = 1.31.

To test the predictions of our Maier–Saupe theory, we study the bulk phase behavior of hard curved spherocylinders with *L*/*D* = 19 and varying particle curvature Ψ using *NPT*-MC simulations, i.e. the number of particles *N*, pressure *P* and temperature *T* are fixed. The resulting phase diagram is shown in Fig. 3b as a function of Ψ and packing fraction *η*. The phase behavior of this athermal lyotropic system is driven by *η* that plays a similar role as the inverse temperature *βϵ* in our Maier–Saupe theory for thermotropic systems. Using this analogy, the comparison of the topology of the theoretical with the computational phase diagram is remarkable. Our simulations confirm the I-N-N_TB_ phase sequence at low particle curvature that transforms into Sm_TSB_ and Sm_SB_ phases upon increasing *η*, as well as a direct I-N_TB_ transition at high particle curvature transforming into Sm_TSB_ and Sm_SB_ phases with increasing density. Our simulations reveal that the splay modulations are accompanied by density modulations in agreement with our predictions from Maier–Saupe theory. In the Supplementary Note 4, we present a mapping of the theoretical and computational phase diagrams using the dependence of the global nematic order parameter *S*_*g*_ on temperature and packing fraction, and we provide a comparison of the orientational order parameters, pitch and conical angles as a function of thermodynamic state, showing quantitative agreement between the predictions of the Maier–Saupe theory and simulations. We note that the main qualitative difference is the absence of the re-entrant Sm_TSB_ phase in the simulated phase diagram. It is important to mention here that due to hysteresis effects and slow equilibration it is impossible to accurately determine the regions of stability and the first- or second-order nature of the phase transitions of the N_TB_, Sm_TSB_, and Sm_SB_ phases in simulations of hard curved spherocylinders. However, the simulations consistently show that the phase transformation from an N_TB_ to an Sm_SB_ phase proceeds via an intermediate Sm_TSB_ phase as twist deformations are gradually replaced by splay deformations of the nematic director field. For example, in Fig. 4 we show typical configurations of an Sm_SB_ phase, an Sm_TSB_ phase, and an N_TB_ phase along an expansion of a system of hard curved spherocylinders of length *L*/*D* = 19 and opening angle Ψ = 1.31 from packing fraction *η* = 0.406 to packing fraction *η* = 0.367. To fully characterise the phases in Fig. 4, we plot the scalar order parameter *S*(*z*), the nematic director field $\hat{{\bf{n}}}(z)$, and the density field *ρ*(*z*) in Supplementary Fig. 12, 13 and 14. In addition, we also plot the polarisation vector $\hat{{\bf{m}}}(z)$ along with the bend distortions in the director field described by the bend vector $\hat{{\bf{b}}}(z)=\hat{{\bf{n}}}\times \left(\nabla \times \hat{{\bf{n}}}\right)/\!\!\parallel \hat{{\bf{n}}}\times \left(\nabla \times \hat{{\bf{n}}}\right)\parallel$. We clearly observe that $\hat{{\bf{m}}}(z)$ is always anti-parallel to $\hat{{\bf{b}}}(z)$, demonstrating that these modulated phases are driven by bend deformations coupled to polar ordering spontaneously arising from the packing constraints of banana-shaped particles.

**Fig. 5:**
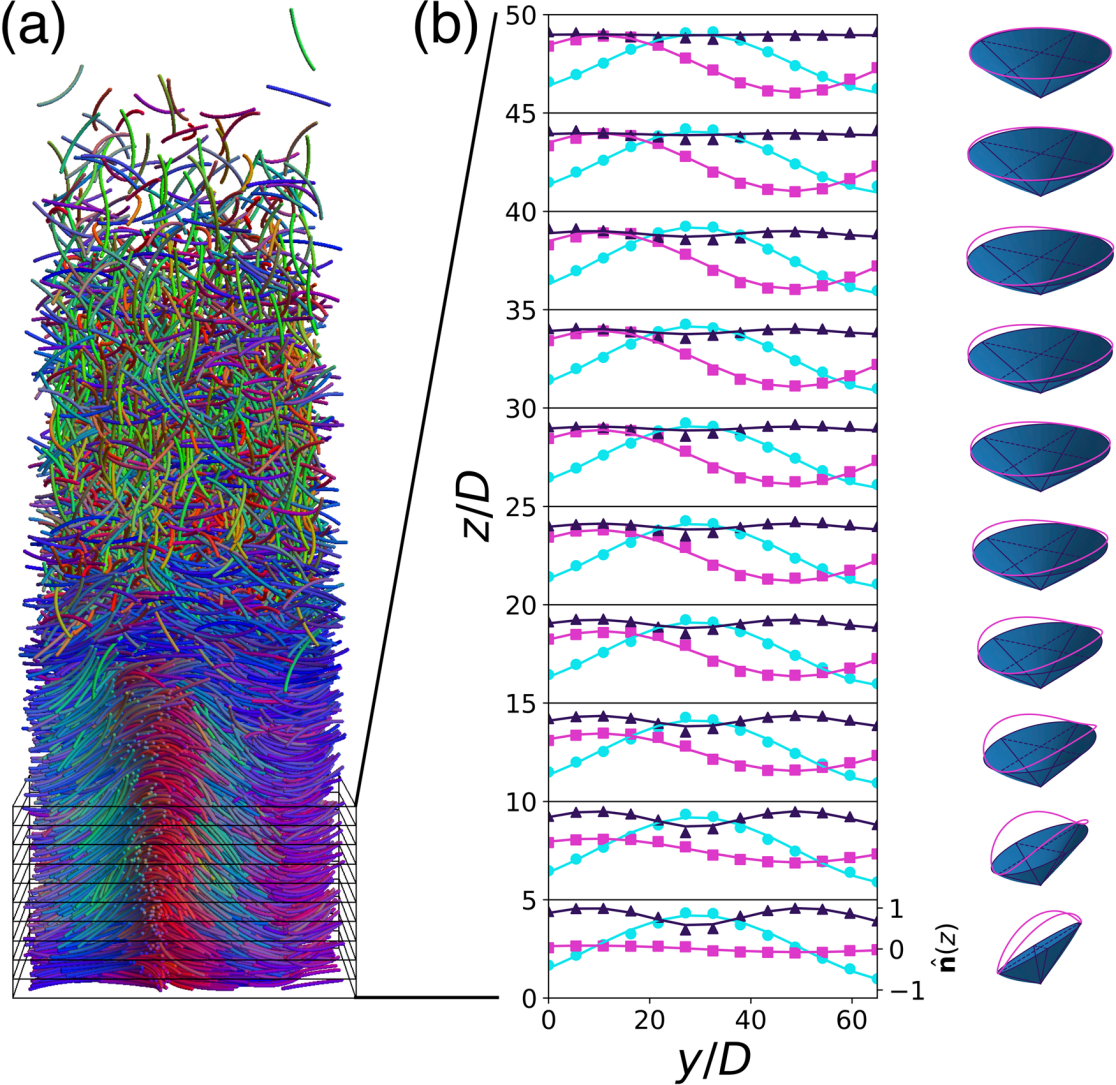
A system of curved rods in gravity. **a** Typical configuration from a simulation on a system of hard curved spherocylinders with an aspect ratio *L*/*D* = 19 and central angle Ψ = 0.99 in a gravitational field along $-\hat{{\bf{z}}}$ with a gravitational length *l*_g_ = 7.5*D* and a hard wall at *z* = 0. The phase sequence I-N-N_TB_-Sm_TSB_-Sm_SB_ is observed from the top to the bottom in the sediment. **b** The *x*-, *y*-, and *z*-components of the nematic director field $\hat{{\bf{n}}}(y)=({n}_{x}(y),{n}_{y}(y),{n}_{z}(y))$ in subsequent slabs of the system of thickness 5*D*, showing a continuous transition from splay-bend deformations to twist-bend deformations with increasing altitude *z*, corresponding to lower pressure and density. The measured values of the nematic director field are denoted by the varying symbols, whereas the lines denote a fit of the simulation data using Eq. (3). The precession cones correspond to the fits of the nematic director field, showing a continuous transition from an N_TB_ phase with a director field precessing around a circular cone at the top of the sediment, via an Sm_TSB_ phase where the precession cone becomes elliptic, towards an Sm_SB_ phase where the elliptical cone reduces to a flat triangle at the bottom of the sample. Source data for (**b**) are provided as a Source Data file.

Finally, we perform simulations on a system of hard curved spherocylinders with *L*/*D* = 19 and Ψ = 0.99 under gravity with a gravitational length *l*_g_ = *k*_B_*T*/*mg* = 7.5*D* parallel to $\hat{{\bf{z}}}$ with a hard wall at the bottom at *z* = 0, *m* denoting the buoyancy mass of the rods and *g* the gravitational acceleration. The resulting configuration, presented in Fig. 5a, shows the full I-N-N_TB_-Sm_TSB_-Sm_SB_ phase sequence in a single system. In particular, we observe a continuous transition from an N_TB_ phase with a director field precessing on a circular cone at the top of the sediment, via an Sm_TSB_ phase where the precession cone becomes elliptic, towards an Sm_SB_ phase where the elliptical cone reduces to a flat triangle at the bottom of the sample. Hence, the transition from the N_TB_ to the Sm_SB_ phase occurs via a continuous flattening of the precession cone of the nematic director field from a right circular cone to an isosceles triangle via a continuous range of elliptical precession cones corresponding to a range of Sm_TSB_ phases. In the Supplementary Note 6, we compare the equation of state obtained by integrating the density profile of the sediment with the equation of state obtained from bulk simulations, showing good agreement.

### Rationalising the topology of modulated liquid crystal phases

Our extensive simulations show that our generalized Maier–Saupe theory effectively predicts, despite its simplicity, the stability of twist-bend, twist-splay-bend, and splay-bend phases of curved spherocylinders. Our microscopic theory is solely based on the tendency of particles to align their particle shape to the local nematic director field $\hat{{\bf{n}}}({\bf{r}})$. To rationalize our findings we calculate the integral curves of the nematic director field $\hat{{\bf{n}}}(z)$, *i.e*. curves **r**(*z*) to which the nematic director $\hat{{\bf{n}}}(z)$ at *z* is tangent, such that ${\bf{r}}^{\prime} (z)=\nu \hat{{\bf{n}}}(z)$ with *ν* a proportionality constant. We thus find

5 $${\bf{r}}(z)={\bf{r}}({z}_{0})+\int_{{z}_{0}}^{z}\,{\text{d}}\,z^{\prime} \nu \hat{{\bf{n}}}(z^{\prime} ),$$

where the integration constant **r**(*z*_0_) = (*x*_0_, *y*_0_, *z*_0_) corresponds to the starting point of an integral curve, such that Eq. (5) actually yields an infinite collection of curves, all identical except for a translation in *x* and *y*.

For a generic twist-splay-bend (TSB) phase the integral in Eq. (5) cannot be evaluated analytically. However, the tendency of particles to align their profiles to the nematic director field at low temperatures or high densities, corresponds to a tendency to match their particle curvature with the curvature of its integral curves. Given a curve **r**(*z*) with tangent ${\bf{r}}^{\prime} (z)=\nu \hat{{\bf{n}}}(z)$ of constant norm $\parallel {\bf{r}}^{\prime} (z)\parallel =\nu$, we can reparametrize it by its arc length as **r**(*s* = *νz*). In this reparametrization the tangent to the curve ${\bf{r}}^{\prime} (s)=(\partial {\bf{r}}(z)/\partial z)z^{\prime} (s)=\hat{{\bf{n}}}(s)$ has unit norm, and we can calculate the curvature of the integral curve as $\kappa (s)=\parallel \hat{{\bf{n}}}^{\prime} (s)\parallel$. Going back to the original parametrization, we obtain $\kappa (z)=\parallel \hat{{\bf{n}}}^{\prime} (z)\parallel\!\! /\nu$. Using this formula, we obtain the following curvature for the integral curve of the nematic director field of a generic TSB phase,

6 $$	\, {\kappa }_{{\rm{TSB}}}(z)=\\ 	\frac{q}{\nu } \, \sqrt{\frac{{\sin }^{2}({\theta }_{{\rm{a}}})-2{\sin }^{2}({\theta }_{{\rm{a}}}){\sin }^{2}({\theta }_{{\rm{b}}})+{\sin }^{2}({\theta }_{{\rm{b}}})+({\sin }^{2}({\theta }_{{\rm{a}}})-{\sin }^{2}({\theta }_{{\rm{b}}}))\cos (2qz)}{2(1-{\sin }^{2}({\theta }_{{\rm{a}}}){\sin }^{2}(qz)-{\sin }^{2}({\theta }_{{\rm{b}}}){\cos }^{2}(qz))}}.$$

**Fig. 6:**
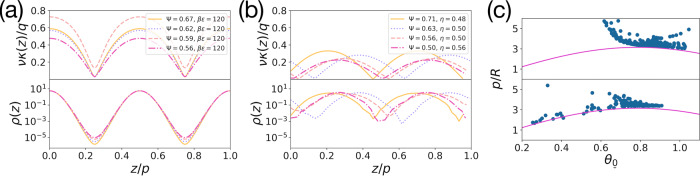
Rationalization of the spatial modulations of the density and the nematic director fields. **a**–**b** Modulations of the curvature *κ*(*z*) of the integral curves of the nematic director field (top), and of the logarithm of the density profile *ρ*(*z*) (bottom) corresponding to minus the effective potential acting on the particles, in splay-bend smectic states (Sm_SB_) predicted by our theory (**a**) and found in Monte Carlo simulations (**b**) for a system of hard curved spherocylinders of various curvatures Ψ and inverse temperatures *βϵ* or packing fractions *η* as labelled. **c** Pitch length *p* versus the conical angle *θ*_0_ of various twist-bend nematic (N_TB_) phases of hard curved spherocylinders of various curvatures Ψ ∈ [0.5, 2] from theory (top) and simulations (bottom), along with the relationship of Eq. (7) (pink line). Source data are provided as a Source Data file.

In the presence of splay deformations, i.e. *θ*_a_ ≠ *θ*_b_, *κ*_TSB_(*z*) is a non-trivial periodic function of *z*. In Fig. 6a–b, we show the curvature *κ*_SB_(*z*) of the integral curves of the nematic director field of various splay-bend (SB) phases with *θ*_a_ = *θ*_0_ and *θ*_b_ = 0 from theory and simulations, along with their density profiles *ρ*(*z*), measuring the probability of finding a particle at *z*. The periodicity of *κ*_SB_(*z*) agrees well with that of $\mathrm{log}\,\rho (z)$, i.e. minus the effective mean-field potential felt by the particles. If the curvature of the integral curves of the nematic director field matches that of the particles, the particles can optimally align their shape to the local nematic director field, resulting into a lower potential energy or higher free volume and thus a higher local density *ρ*(*z*), whereas geometric frustration arises when the curvature of the integral curve deviates from that of the particles, leading to a higher potential energy or lower free volume and hence a lower local density. We thus conclude that the presence of periodic density modulations in TSB and SB phases, and hence Sm_TSB_ and Sm_SB_ phases, can be explained geometrically by the coupling of particle curvature to the non-uniform bend deformations of the director field, but also by the coupling of density to splay deformations^27,28^.

On the other hand, in the case of an N_TB_ phase the translational symmetry of the elastic deformations is preserved, see Supplementary Note 2, and we expect the curvature of the integral curves of the nematic director field to be uniform. Intriguingly, for N_TB_ phases with *θ*_a_ = *θ*_b_ = *θ*_0_ the integration of Eq. (5) can be carried out explicitly, yielding the following expression ${{\bf{r}}}_{{\rm{TB}}}(z)={{\bf{r}}}_{{\rm{TB}}}({z}_{0})+\frac{\nu }{q}\sin {\theta }_{0}[\sin (qz)-\sin (q{z}_{0})]\,{\bf{i}}-\frac{\nu }{q}\sin {\theta }_{0}[\cos (qz)-\cos (q{z}_{0})]\, {\bf{j}}+\nu \cos {\theta }_{0}[z-{z}_{0}]\, {\bf{k}}$ for the integral curve of the nematic director field, *i.e*. a helix of period $p\nu \cos {\theta }_{0}$. Hence, we can impose that the integral curve has the same pitch *p* as the N_TB_ phase — or, equivalently, that ${\bf{r}}(z)\cdot \hat{z}=z$ — by setting $\nu =1/\cos {\theta }_{0}$. As expected, the curvature of Eq. (6) reduces to a uniform value ${\kappa }_{{\rm{TB}}}(z)=(q/\nu )\sin ({\theta }_{0})=q\sin (2{\theta }_{0})/2$ independent of *z*. Using the conjecture that particles tend to match their curvature with the one of the integral curves of the nematic director field, we impose *κ*_TB_ = 1/*R*, and obtain the simple relation

7 $$p=\pi R\sin (2{\theta }_{0})$$

between the pitch *p* and conical angle *θ*_0_ of an N_TB_ phase and the particle curvature *R*. In Fig. 6c we test this simple relation against N_TB_ phases from theory and simulations, finding that it describes the data remarkably well without any fit parameter. The most significant deviation is found for the theoretically predicted N_TB_ states close to the I-N phase transition, where the pitch increases significantly across a small temperature range^24^, a behavior not captured by Eq. (7) and our simulations. We remark here that the absence of an increase in the pitch in simulations may be caused by the finite size of the simulation box and the periodic boundary conditions. However, larger simulation boxes are beyond the limits of our computational resources. Figure 6c shows that the conical angle and the pitch vary in the range *θ*_0_ ∈ [0.2, 0.9], and *p*/*R* ∈ [1, 5]. We thus find that the pitch length *p* is on the order of the radius of curvature *R* of a particle independent of *L*, which can be used as a simple design rule and is consistent with the small pitch lengths observed for thermotropic bent-core mesogens^1–12^. We remark here that this phase is also called a polar twisted nematic instead of an N_TB_ phase only because of its small pitch length^29,30^. In this work we prefer to classify the liquid crystal phases according to their symmetries. We also note that the smectic layer spacing *λ* = *p*/2 varies as *λ*/*L* ∈ [0.32, 4.16]. Hence, the unidentified Sm_X_ phase with a smectic layer spacing of *λ* ≃ 0.5*L* may perhaps be a Sm_TSB_ or Sm_SB_ phase^21^.

## Discussion

We introduce a generic nematic N_TSB_ phase with twist, splay, and bend modulations in the director field which reduces to N, N^*^, N_TB_, and N_SB_ phases in limiting cases. We use the nematic director field of this N_TSB_ phase as a variational ansatz to develop a simple but comprehensive variational Maier–Saupe theory of periodically deformed nematic and smectic phases. We exploit this mean-field theory to predict the phase behavior of curved rods as a function of thermodynamic state and microscopic details, and find excellent agreement with simulations on hard curved spherocylinders. To characterise the symmetry and local structure of these modulated phases, we measure the scalar order parameter *S*(*z*), nematic director field $\hat{{\bf{n}}}(z)$, polarisation vector $\hat{{\bf{m}}}(z)$, and density field *ρ*(*z*) in simulations. We observe that the polarization is always anti-parallel to the director of the bend deformations, demonstrating that polarization and bend deformation are coupled by the flexo-electric effect originally proposed by Meyer^13^. We conclude that the mechanism behind these spatially modulated phases is the spontaneous polar ordering and bend deformations arising from the packing efficiency of curved particles. Our key findings are (1) that the N_TB_ transforms into a Sm_SB_ phase (or vice versa) via a gradual squeezing (or opening) of the precession cone, passing through an intermediate Sm_TSB_ phase, and (2) that the elusive N_SB_ phase is unstable with respect to the density-modulated Sm_SB_ phase. Accurate free-energy calculations in computer simulations are required to determine the thermodynamic stability of the Sm_TSB_ phase and the nature of the N_TB_, Sm_TSB_, and Sm_SB_ phase transitions in the bulk phase diagram of hard curved spherocylinders.

Moreover, the agreement between the phase diagram determined by our simple mean-field theory for a thermotropic system and the one obtained from simulations for a lyotropic system, not only demonstrates the predictive power of our simple variational Maier–Saupe theory, but also provides strong evidence that the particle curvature is the driving force behind the topology of the spatial director-field modulations in the N_TB_, Sm_TSB_, and Sm_SB_ phases. To rationalize this finding, we calculate the integral curves of the nematic director field. We show that the curvature of these integral curves is periodic in the case of Sm_TSB_ and Sm_SB_ phases, and that the coupling of particle curvature to the non-uniform curvature of the director field leads to periodic density modulations. In the case of N_TB_ phases, we derive an explicit expression for the uniform curvature of the nematic director field integral curves. By matching this curvature with that of the particles, we derive a simple relationship between the pitch and conical angle of the N_TB_ phase and the microscopic particle curvature. We verify this simple relationship using theory and simulations.

In conclusion, our variational ansatz for a twist-splay-bend phase is a powerful tool for predicting, understanding and rationalising spatially modulated liquid crystal phases. Exploiting the generality of this variational ansatz in a generalized Maier–Saupe theory enabled us to predict not only the stability of twist-bend and splay-bend phases, but also the orientational order parameters, pitch and conical angle as a function of the thermodynamic state and microscopic details of the particles, see Supplementary Note 5. This variational ansatz can also be exploited in Landau–de Gennes and Oseen–Frank theories of spatially modulated phases. Further improvements of the Maier–Saupe theory such as introducing biaxiality^31^, extending the description from prolate to oblate liquid crystals, or generalizing the variational ansatz for spatial modulations from 1D to 2D and 3D to describe polar blue phases^32^, may lead to a generic theoretical framework of modulated liquid crystal phases.

### Methods

#### Variational Maier–Saupe theory

To solve our generalized Maier–Saupe theory, we minimize the free energy in Eq. (4) with respect to the variational parameters of the director- and density-field. Calculating the free-energy difference per particle *β*Δ*F*/*N* is trivial except for the partition function *Q*, i.e. an integral of the form

8 $$I= 	\,\int \,{\text{d}}\,{{\Omega }}\int_{0}^{p}\,{\text{d}}\,Z\ f(Z,{{\Omega }})\\ =	 \int_{0}^{2\pi }\,{\text{d}}\alpha \int_{0}^{\pi }\sin \beta {\text{d}}\beta\,\int_{0}^{2\pi }{\text{d}}\gamma \int_{0}^{p}{\text{d}}\,Z\ f(Z,{{\Omega }}),$$

with $f(Z,{{\Omega }})=\exp [-\beta U(Z,{{\Omega }})]$. We assume that the function *f*(*Z*, Ω) is a periodic function in *Z* with period *p*. To evaluate the integrals of the form (8), we first transform the original space of integration to a 4-dimensional hypercube [−1, 1]^4^ using the transformation $\alpha =\pi (\xi +1),\cos \beta =\eta ,\gamma =\pi (\mu +1),Z=\frac{p}{2}(\psi +1)$ with Jacobian ∣*J*∣ = *pπ*^2^/2. The integral in Eq. (8) becomes

9 $$I=\int_{-1}^{1}\,{\text{d}}\xi \int_{-1}^{1}{\text{d}}\eta \int_{-1}^{1}{\text{d}}\mu \int_{-1}^{1}{\text{d}}\,\psi \ \frac{p{\pi }^{2}}{2}f(Z(\psi ),{{\Omega }}(\xi ,\eta ,\mu )),$$

which we solve using an ${\mathcal{N}}$-points Gauss-Legendre quadrature. If {*ξ*_*i*_}, {*η*_*i*_}, {*μ*_*i*_}, and {*ψ*_*i*_} are the ${\mathcal{N}}$ node points in [−1, 1], *i.e*. the ${\mathcal{N}}$ roots of the ${\mathcal{N}}$-th Legendre polynomial, and {*w*_*ξ*,*i*_}, {*w*_*η*,*i*_}, {*w*_*μ*,*i*_}, and {*w*_*ψ*,*i*_} are the associated weights, we can approximate the integral *I* of Eq. (9) by

10 $$I\approx \mathop{\sum}\limits_{i = 1}^{{\mathcal{N}}}\mathop{\sum }\limits_{j = 1}^{{\mathcal{N}}}\mathop{\sum }\limits_{k = 1}^{{\mathcal{N}}}\mathop{\sum }\limits_{l = 1}^{{\mathcal{N}}}\ {w}_{\xi ,i}{w}_{\eta ,j}{w}_{\mu ,k}{w}_{\psi ,l}\frac{p{\pi }^{2}}{2}f(Z({\psi }_{l}),{{\Omega }}({\xi }_{i},{\eta }_{j},{\mu }_{k})).$$

Using the approximation of Eq. (10) and $f(Z(\psi ),{{\Omega }}(\xi ,\eta ,\mu ))=\exp [-\beta U(Z(\psi ),{{\Omega }}(\xi ,\eta ,\mu ))]$, we evaluate the partition function in Eq. (4) using an ${\mathcal{N}}$ = 32 points Gauss-Legendre integration.

Once *Q* is calculated, we minimize *β*Δ*F*/*N* at given *βϵ* in the 6-dimensional space of parameters *S*, *τ*, *n*, *θ*_a_, *θ*_b_ and *q* = 2*π*/*p* by means of a Covariance Matrix Adaptation Evolution Strategy (CMA-ES) using the Python library in https://pypi.org/project/cma/.

#### Monte Carlo simulations

We study the phase behavior of a system consisting of hard curved spherocylinders using Monte Carlo simulations in the *NPT* ensemble, *i.e*. at fixed number of particles *N*, pressure *P* and temperature *T*. We employ an orthorhombic simulation box of sides *L*_*x*_, *L*_*y*_, and *L*_*z*_ and apply periodic boundary conditions. We perform a sequence of MC cycles consisting of *N* + 1 MC moves. Each MC move consists of a particle move with probability ~*N*/(*N* + 1), and a volume move with probability ~1/(*N* + 1). In a particle move, a random roto-translation of a randomly picked particle is proposed and accepted if it does not generate overlaps with other particles. In a volume move, a random variation of a random side of the simulation box is proposed, and the system is compressed or expanded accordingly. If the compression/expansion does not generate overlaps between the particles, the move is accepted with a probability

11 $${\rm{acc}}(V\to V^{\prime} )=\min \left(1,{\left(\frac{V^{\prime} }{V}\right)}^{N}{e}^{-\beta P{{\Delta }}V}\right),$$

where ${{\Delta }}V=V^{\prime} -V$ denotes the change in volume. We perform simulations on a system of *N* = 2048 hard curved spherocylinders, and initialize all simulations from a nematic configuration. We measure a wide set of observables during the simulation, like density, uniaxial nematic order parameters, smectic order parameters, etc. When the system reaches equilibrium as monitored from the observables, we characterize the system’s configuration. We find that 10^8^ MC cycles are typically sufficient for equilibration.

To study a system of hard curved spherocylinders in a gravitational field, we perform Monte Carlo simulations in an *NVT* ensemble, i.e. we fix the number of particles *N* = 8192, volume *V*, and temperature *T*. We implement a hard wall at the bottom at *z* = 0 and apply periodic boundary conditions in the *x* − and *y* − direction. Each MC cycle consists of *N* attempts to randomly rotate and translate a randomly selected particle. If a particle roto-translation leads to an overlap with one of the particles or with the hard wall, the move is rejected, otherwise it is accepted with a probability

12 $${\rm{acc}}(z\to z^{\prime} )=\min (1,{e}^{-\beta {{\Delta }}{U}_{{\rm{g}}}}),$$

with ${{\Delta }}{U}_{{\rm{g}}}=(z^{\prime} -z)/{l}_{{\rm{g}}}$ the change in potential energy due to gravity, *z* and $z^{\prime}$ the old and new *z*-coordinate of the displaced particle, and *l*_g_ the gravitational length of the system.

## Supplementary information

Supplementary Information

Peer Review File

## Data Availability

The source data from Figs. 3, 5b, 6, Supplementary Figs. 1, 2,  3,  7, 8,  9,  11,  12,  13,  14, and  15 are provided in the source data file. All the other relevant data associated with this research is available upon request. Source data are provided with this paper.

## Source data

Source Data

## Code availability

The simulation and analysis codes associated with this research are available upon request.
