# Supplementary material for: A generalized density-modulated twist-splay-bend phase of banana-shaped particles
Source: Nat Commun. 2021 Apr 12;12:2157. doi: 10.1038/s41467-021-22413-8 (PMC8041804; doi:10.1038/s41467-021-22413-8)
Supplement: Supplementary file 1 — Supplementary Information [file 41467_2021_22413_MOESM1_ESM.pdf]

Supplementary Information

A generalized density-modulated twist-splay-bend phase of banana-shaped particles

Massimiliano Chiappini[1,*] and Marjolein Dijkstra[1,†]

[1] *Soft Condensed Matter, Debye Institute for Nanomaterials Science, Department of Physics, Utrecht University, Princetonplein 1, 3584 CC Utrecht , The Netherlands.*

[*] *e-mail: m.chiappini@uu.nl*

[†] *e-mail: m.dijkstra@uu.nl*

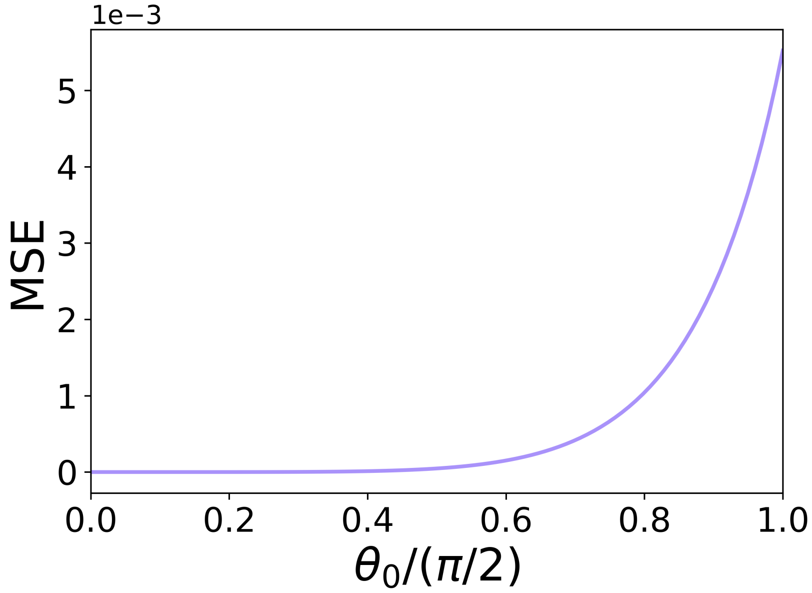

Supplementary Figure 1: Mean square difference with Dozov's splay-bend nematic director field. Mean square difference MSE between our expression $\hat{\mathbf{n}}_{\mathrm{TSB}}(z|\theta_0, 0, q)$ and Dozov's expression $\hat{\mathbf{n}}_{\mathrm{SB}}(z)$[1] over a full pitch length p as a function of conical angle θ_0. Source data are provided as a Source Data file.

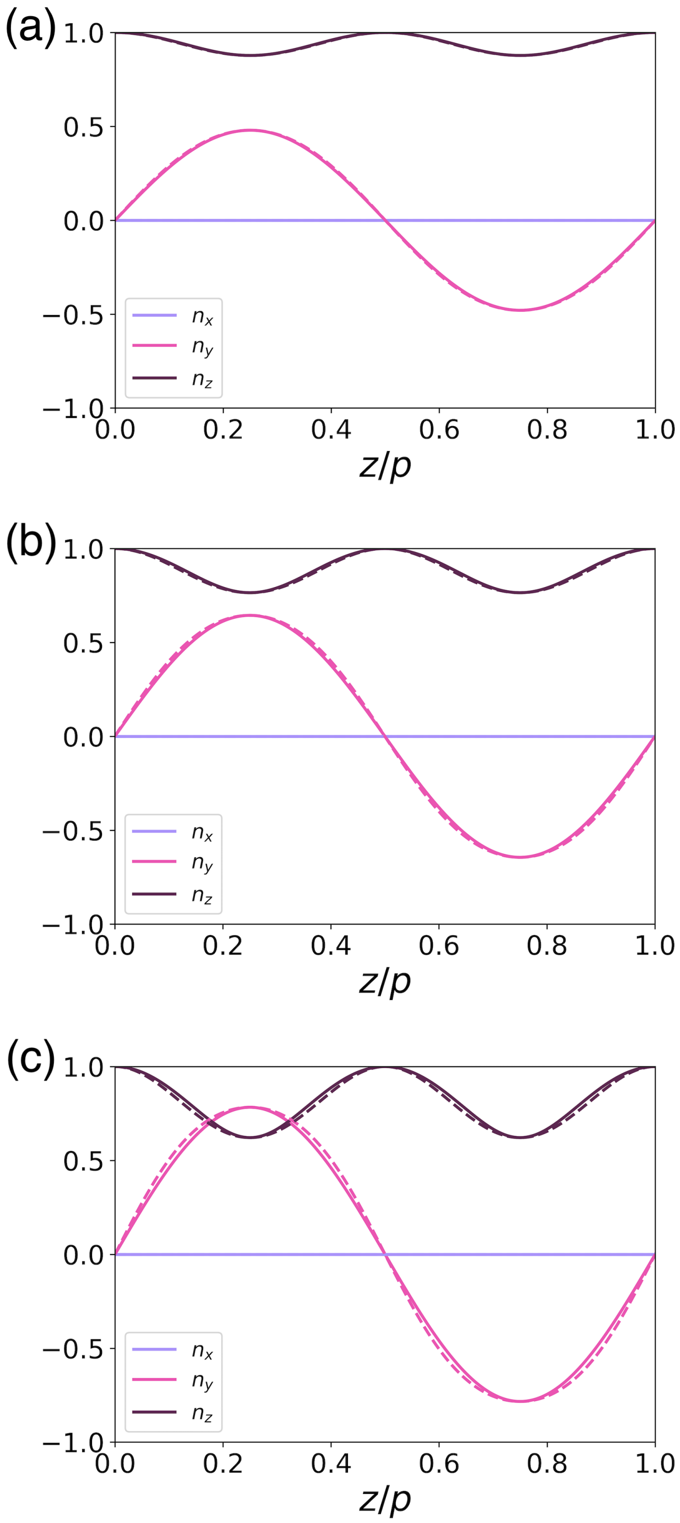

Supplementary Figure 2: Comparison with Dozov's splay-bend nematic director field. Comparison of the x-, y-, and z-components of our expression for the nematic director field $\hat{\mathbf{n}}_{\mathrm{TSB}}(z|\theta_0, 0, q)$ (full lines) and Dozov's expression $\hat{\mathbf{n}}_{\mathrm{SB}}(z)$[1] (dashed lines) of an $\mathrm{N_{SB}}$ phase as a function of z over a full pitch length p for conical angle $\theta_0 = 0.5$ (a), 0.7 (b), and 0.9 (c). Source data are provided as a Source Data file.

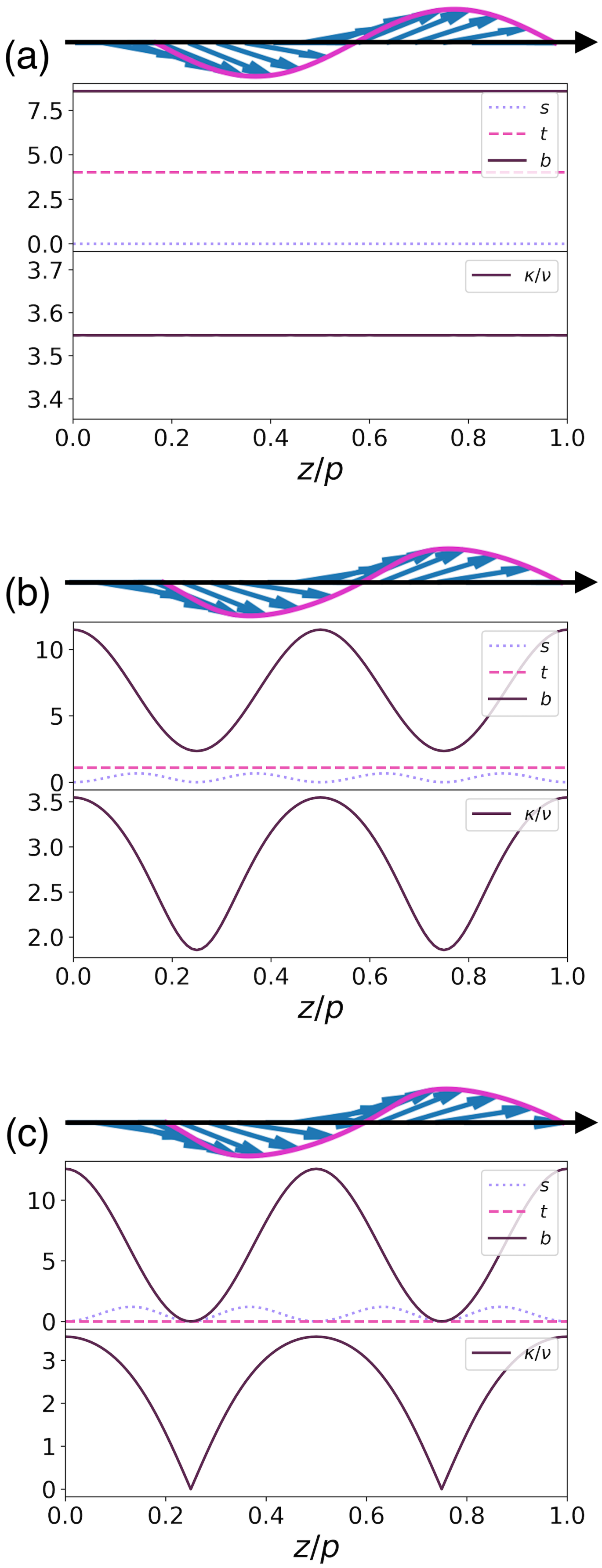

Supplementary Figure 3: Splay, twist, and bend deformations in $\mathrm{N_{TB}}$**,** $\mathrm{N_{TSB}}$**, and** $\mathrm{N_{SB}}$ **phases.** Splay (s), twist (t), and bend (b) deformations as calculated via Supplementary Equation 1 (top panel) and curvature κ/ν of the integral curve of the nematic director field (bottom panel, see main text) of an (a) $\mathrm{N_{TB}}$, (b) $\mathrm{N_{TSB}}$, and (c) $\mathrm{N_{SB}}$ phase. Source data are provided as a Source Data file.

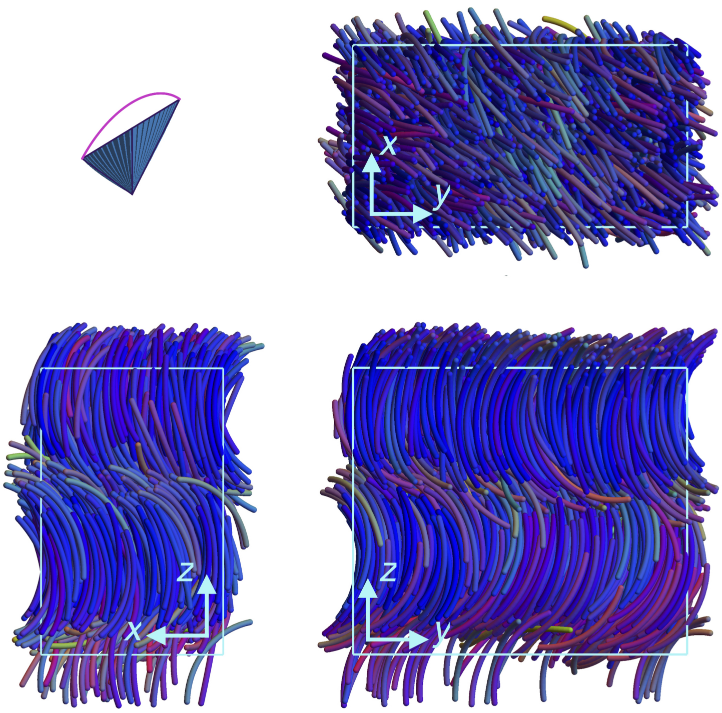

Supplementary Figure 4: Splay-bend smectic phase. Typical configuration of an $\mathrm{Sm_{SB}}$ phase with conical angle $\theta_\mathrm{a} \sim 0.76$ and $\theta_\mathrm{b} \sim 0$ at packing fraction $\eta = 0.394$ (box size $55.5D \times 30.3D \times 47.7D$) obtained from simulations of hard curved spherocylinders of length $L/D = 19$ and opening angle $\Psi = 1.31$.

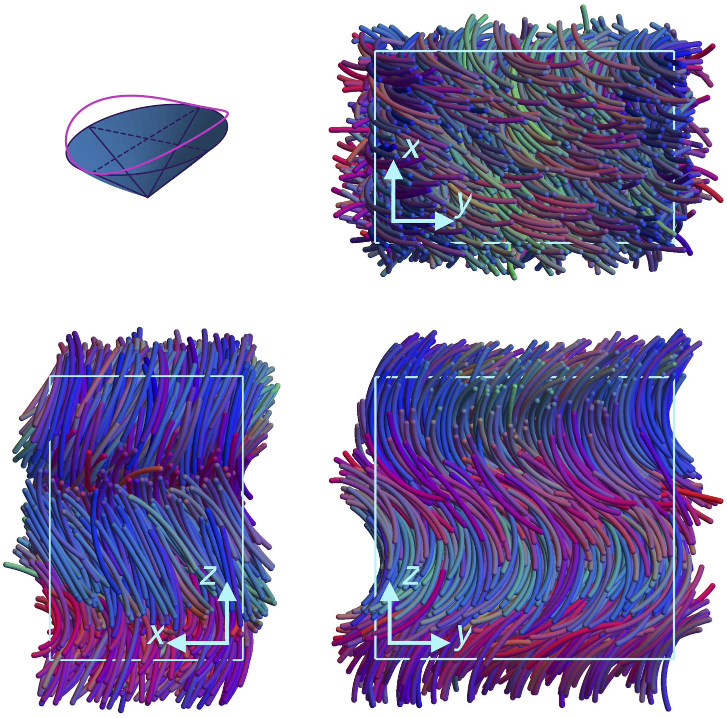

Supplementary Figure 5: Twist-splay-bend smectic phase. Typical configuration of an $\mathrm{Sm_{TSB}}$ phase with conical angle $\theta_\mathrm{a} \sim 0.87$ and $\theta_\mathrm{b} \sim 0.51$ at packing fraction $\eta = 0.371$ (box size $52.0D \times 33.4D \times 49.1D$) obtained from simulations of hard curved spherocylinders of length $L/D = 19$ and opening angle $\Psi = 1.31$.

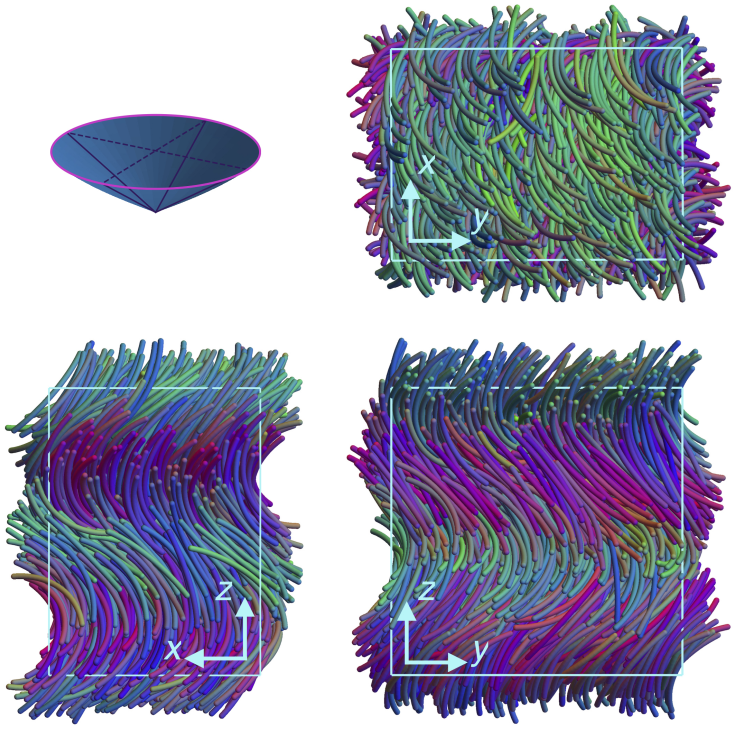

Supplementary Figure 6: Twist-bend nematic phase. Typical configuration of an N_{TB} phase with conical angle $\theta_a \sim \theta_b \sim 0.83$ at packing fraction $\eta = 0.354$ (box size $49.9D.3D \times 49.3D$) obtained from simulations of hard curved spherocylinders of length $L/D = 19$ and opening angle $\Psi = 1.31$.

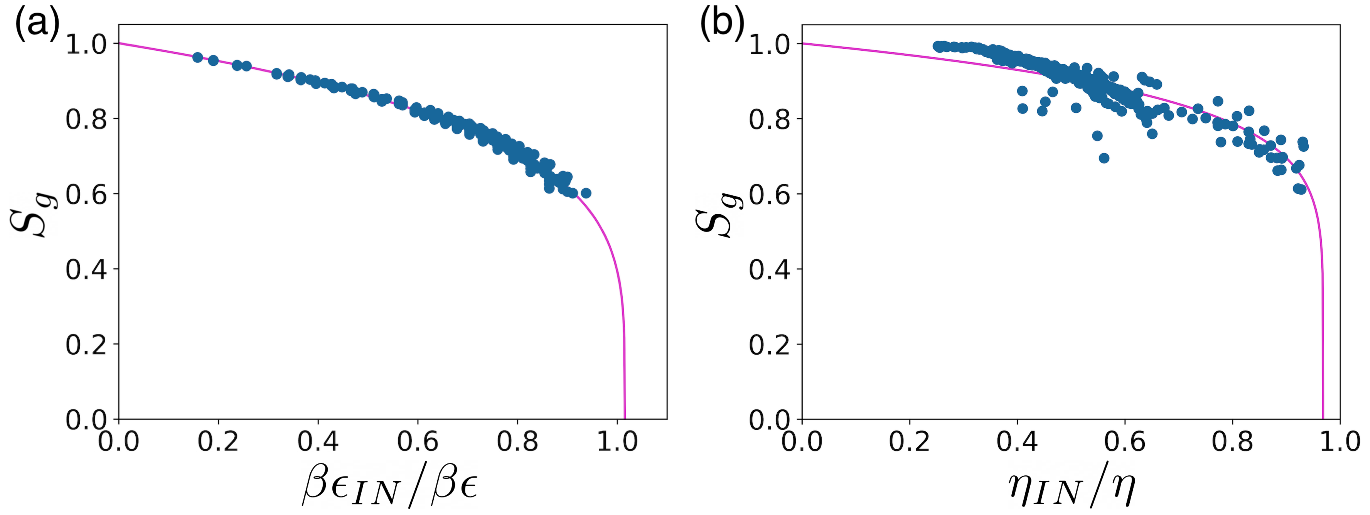

Supplementary Figure 7: Fits of the global nematic order parameter from theory and simulations. The global nematic order parameter S_g as a function of $\beta\epsilon_{IN}/\beta\epsilon$ from theory (a), and as a function of η_{IN}/η from simulations (b). The fits using Supplementary Equations 2 and 3, respectively, are denoted by the pink lines, yielding the values $\alpha_{th} = 0.98$ and $\gamma_{th} = 0.22$, and $\alpha_{sim} = 1.03$ and $\gamma_{sim} = 0.14$. Source data are provided as a Source Data file.

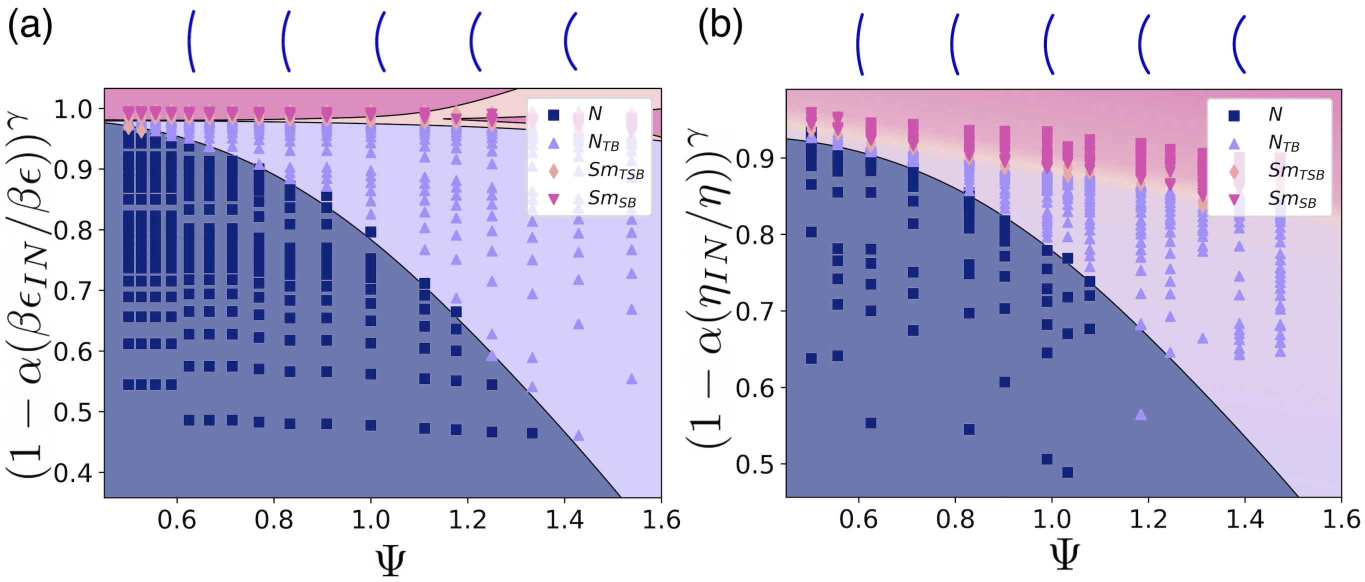

Supplementary Figure 8: Theoretical and computational phase diagrams rescaled as a function of the global nematic order parameter. Phase diagrams from theory (a) and simulations (b) as a function of the global nematic order parameter S_{g} as calculated from Supplementary Equations 2 and 3, with $\alpha_{\mathrm{th}} = 0.98$, $\gamma_{\mathrm{th}} = 0.22$, $\alpha_{\mathrm{sim}} = 1.03$ and $\gamma_{\mathrm{sim}} = 0.14$ obtained from the fits in Supplementary Figure 7 along with $\beta\epsilon_{\mathrm{IN}}(\Psi)$ and $\eta_{\mathrm{IN}}(\Psi)$ obtained from the Maier-Saupe theory and simulations. Source data are provided as a Source Data file.

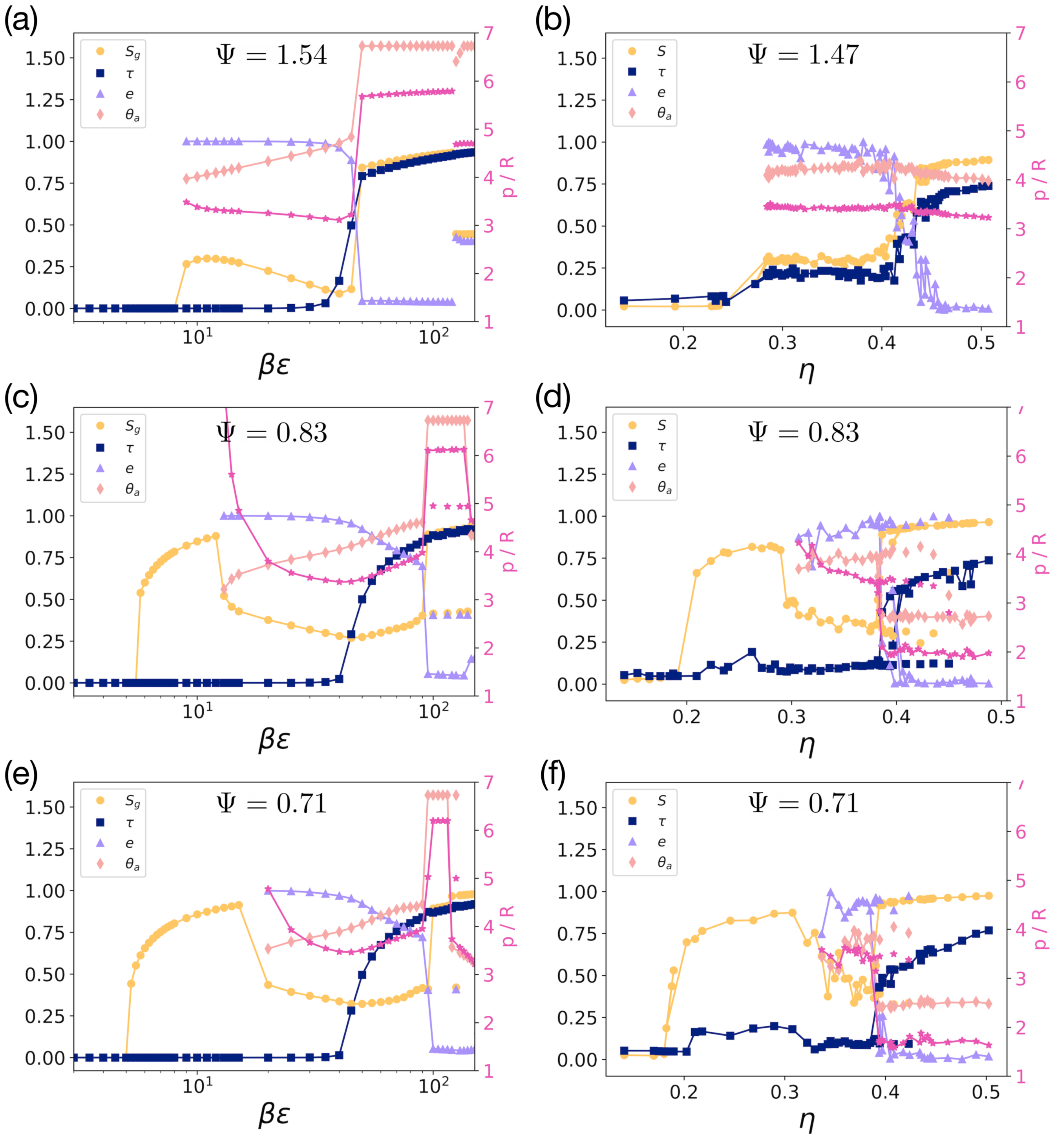

Supplementary Figure 9: Comparison of equations of state from theory and simulations. Comparison of the global nematic order parameter S_{g}, smectic order parameter τ, ellipticity $e = \theta_{\mathrm{b}}/\theta_{\mathrm{a}}$, conical angle θ_{a}, and pitch length p (right axis) for a system of curved spherocylinders with opening angles Ψ as labeled obtained from the Maier-Saupe theory (left) and simulations (right), as a function of inverse temperature $\beta\epsilon$ and packing fraction η, respectively. Source data are provided as a Source Data file.

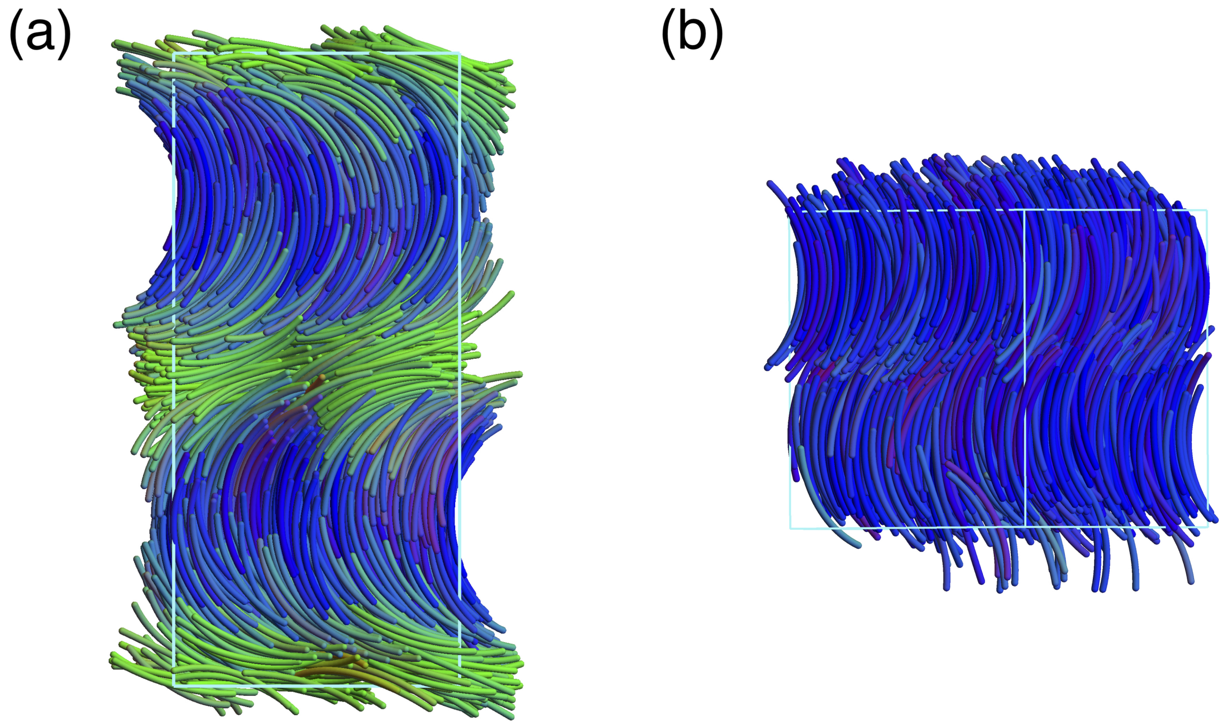

Supplementary Figure 10: Comparison of a $\mathrm{Sm_{SB}}$ **state with conical angle** $\theta_\mathrm{a} \sim \pi/2$ **and a typical** $\mathrm{Sm_{SB}}$ **state from our simulations.** (a) Typical configuration from a MC simulation of an $\mathrm{Sm_{SB}}$ state with conical angle $\theta_\mathrm{a} \sim \pi/2$ of hard curved spherocylinders of length $L/D = 19$ and opening angle $\Psi = 1.05$ at packing fraction $\eta = 0.41$. Our Maier-Saupe theory commonly predicts this kind of $\mathrm{Sm_{SB}}$ state, characterised by full splay/bend swipes, which indeed proves to be mechanically stable in MC simulations. However, compression runs from an N and $\mathrm{N_{TB}}$ states of hard cuved spherocylinders with $L/D = 19$ and opening angle $\Phi = 1.03$ in relatively small simulation boxes result into an $\mathrm{Sm_{SB}}$ phase with a small pitch p and conical angle θ_a as shown in (b), supposedly to preserve continuity in the pitch length and conical angle.

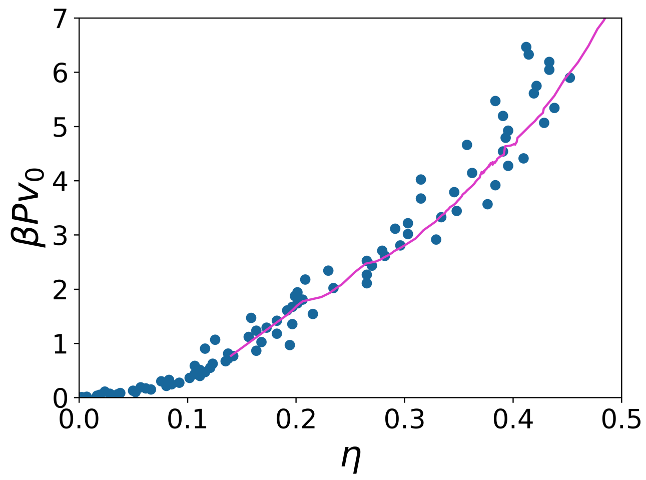

Supplementary Figure 11: Equations of state in bulk and in sedimentation. Comparison of the equations of state, i.e. pressure $\beta P v_0$ versus packing fraction η with v_0 the particle volume, of hard curved spherocylinders of aspect ratio $L/D = 19$ and opening angle $\Psi = 0.99$ as obtained from bulk simulations and from integrating the density profile of a system subject to gravity with a gravitational length $l_\mathrm{g} = 7.5D$ (blue symbols). Source data are provided as a Source Data file.

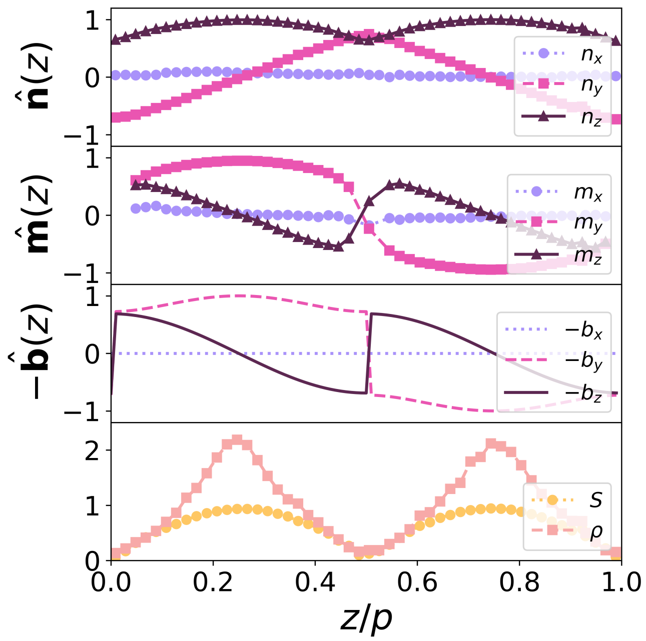

Supplementary Figure 12: Nematic director field, polarity, and bend deformations in a $\mathrm{Sm_{SB}}$ phase. Comparison of the measured nematic director field $\hat{\mathbf{n}}(z)$, the normalized polarization vector $\hat{\mathbf{m}}(z)$, the bend distortions of the director as described by the normalized bend vector $\hat{\mathbf{b}}(z)$ calculated from the theoretical nematic director field $\hat{\mathbf{n}}(z)$ of Equation 3, and the scalar order parameter $S(z)$ and the probability $\rho(z)$ of finding a particle at z for the $\mathrm{Sm_{SB}}$ phase of hard curved spherocylinders of aspect ratio $L/D = 19$ and opening angle $\Psi = 1.31$ reported in Figure 4a and in Supplementary Figure 4. Source data are provided as a Source Data file.

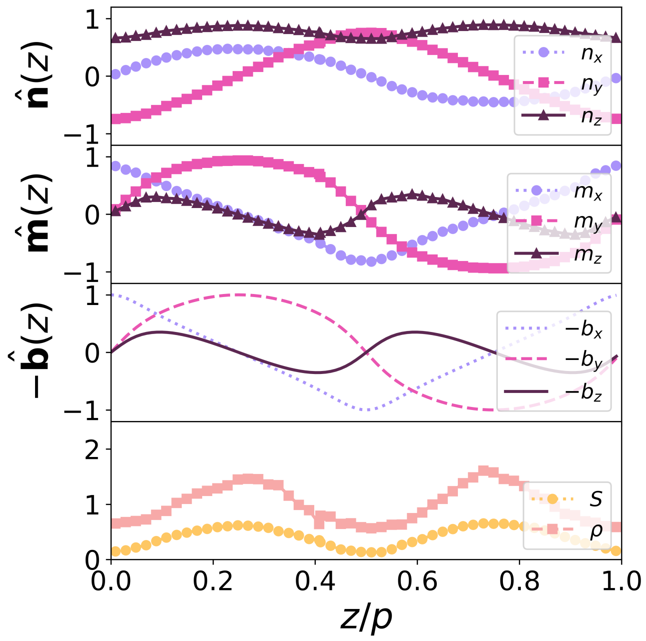

Supplementary Figure 13: Nematic director field, polarity, and bend deformations in a $\mathrm{Sm_{TSB}}$ phase. Comparison of the measured nematic director field $\hat{\mathbf{n}}(z)$, the normalized polarization vector $\hat{\mathbf{m}}(z)$, the bend distortions of the director as described by the normalized bend vector $\hat{\mathbf{b}}(z)$ calculated from the theoretical nematic director field $\hat{\mathbf{n}}(z)$ of Equation 3, and the scalar order parameter $S(z)$ and the probability $\rho(z)$ of finding a particle at z for the $\mathrm{Sm_{TSB}}$ phase of hard curved spherocylinders of aspect ratio $L/D = 19$ and opening angle $\Psi = 1.31$ reported in Figure 4b and in Supplementary Figure 5. Source data are provided as a Source Data file.

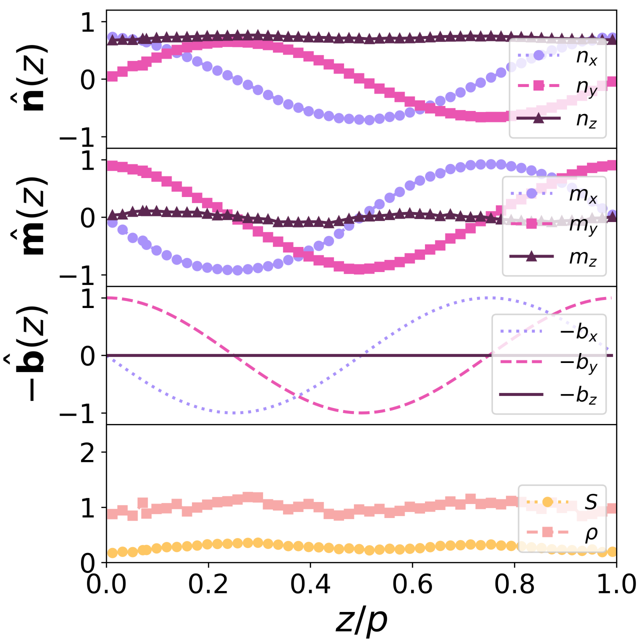

Supplementary Figure 14: Nematic director field, polarity, and bend deformations in a $\mathrm{N_{TB}}$ phase. Comparison of the measured nematic director field $\hat{\mathbf{n}}(z)$, the normalized polarization vector $\hat{\mathbf{m}}(z)$, the bend distortions of the director as described by the normalized bend vector $\hat{\mathbf{b}}(z)$ calculated from the theoretical nematic director field $\hat{\mathbf{n}}(z)$ of Equation 3, and the scalar order parameter $S(z)$ and the probability $\rho(z)$ of finding a particle at z for the $\mathrm{N_{TB}}$ phase of hard curved spherocylinders of aspect ratio $L/D = 19$ and opening angle $\Psi = 1.31$ reported in Figure 4c and in Supplementary Figure 6. Source data are provided as a Source Data file.

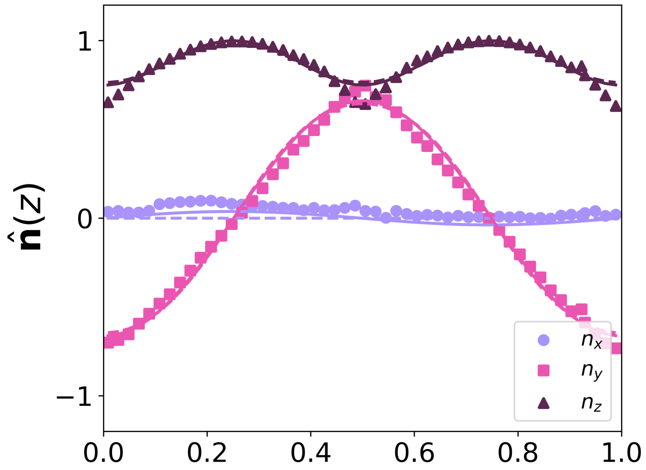

Supplementary Figure 15: Fits of the nematic director field of a $\mathrm{Sm_{SB}}$ with our and Dozov's expression. x, y, and z components of the nematic director field $\hat{\mathbf{n}}(z)$ of the $\mathrm{Sm_{SB}}$ phase of hard curved spherocylinders of aspect ratio $L/D = 19$ and opening angle $\Psi = 1.31$ reported in Figure 4c and in Supplementary Figure 4 fitted with our (solid lines) and Dozov's (dashed lines) expressions for the nematic director field of a splay-bend nematic phase. Source data are provided as a Source Data file.

Supplementary Note 1: Nematic director field of the $\mathrm{N_{SB}}$ phase

In the main text we define a $\mathrm{N_{SB}}$ phase as a special case of the more general $\mathrm{N_{TSB}}$ phase with $\theta_\mathrm{b} = 0$, yielding the nematic director field $\hat{\mathbf{n}}_\mathrm{TSB}(z|\theta_\mathrm{a} = \theta_0, \theta_\mathrm{b} = 0, q) = \sin(\theta_0)\sin(qz)\,\mathbf{j} + \sqrt{1-\sin^2(\theta_0)\sin^2(qz)}\,\mathbf{k}$. The commonly accepted nematic director field of a $\mathrm{N_{SB}}$ phase is the one presented by Dozov in [1], *i.e.* $\hat{\mathbf{n}}_\mathrm{SB}(z) = \sin(\theta_0\sin(qz))\,\mathbf{j} + \cos(\theta_0\sin(qz))\,\mathbf{k}$. Although being different, the two expressions result to be indistinguishable for small conical angles θ_0.

To illustrate this, we first consider the y-component of $\hat{\mathbf{n}}_\mathrm{TSB}(z|\theta_0, 0, q)$, *i.e.* $\sin(\theta_0)\sin(qz)$. In the small angle approximation $\sin\theta_0 \approx \theta_0$, and $\sin(\theta_0)\sin(qz) \approx \theta_0\sin(qz)$, which is smaller or equal than θ_0 since $|\sin(qz)| \leq 1$. Hence, for small conical angles $\theta_0\sin(qz) \approx \sin(\theta_0\sin(qz))$, thereby recovering Dozov's expression for the y-component. The derivation for the z-component is now straightforward. Using $\sin\theta_0\sin(qz) \approx \sin(\theta_0\sin(qz))$ for small conical angles, we find $\sqrt{1-\sin^2(\theta_0)\sin^2(qz))} \approx \sqrt{1-\sin^2(\theta_0\sin(qz))} \approx \cos(\theta_0\sin(qz))$, thereby recovering Dozov's expression for the z-component.

In Supplementary Figure 1 we show the mean square difference MSE between our and Dozov's expression for the nematic director field of a $\mathrm{N_{SB}}$ phase as a function of conical angle θ_0, showing that although the difference increases with θ_0 it remains small for all $\theta_0 \in [0, \pi/2]$, i.e. MSE $< 5 \times 10^{-3}$. In Supplementary Figure 2 we show explicitly a comparison of the two expressions of the nematic director field over a full pitch length for conical angle $\theta_0 = 0.5, 0.7$, and 0.9.

Both Dozov's expression and our expression can be used in an Oseen-Frank theory or a Maier-Saupe theory as variational ansatzes for the nematic director field with the conical angle and pitch length as the variational parameters. Minimizing the free energy with respect to these variational parameters gives the equilbrium phase. Although we cannot exclude that a full minimization of the free energy may give a slightly different nematic director field with an even lower free energy, we note that the nematic director field of the SB phases measured in our simulations are well-fitted by both expressions (see Supplementary Figure 15).

Supplementary Note 2: Splay, twist, and bend deformations in the $\mathrm{N_{TSB}}$ phase

The deformations of splay, twist, and bend of the nematic director field $\hat{\mathbf{n}}(\mathbf{r})$ are given by

$$\begin{aligned} s &= (\nabla\cdot\hat{\mathbf{n}})^2 \\ t &= (\hat{\mathbf{n}}\cdot(\nabla\times\hat{\mathbf{n}}))^2 \\ b &= (\hat{\mathbf{n}}\times(\nabla\times\hat{\mathbf{n}}))^2\,. \end{aligned} \tag{1}$$

In Supplementary Figure 3 (top panels) we report the splay, twist, and bend deformations of the nematic director field of exemplary $\mathrm{N_{TB}}$, $\mathrm{N_{TSB}}$, and $\mathrm{N_{SB}}$ phases, calculated via Supplementary Equation 1. The $\mathrm{N_{TB}}$ phase is characterised by a zero splay and by uniform twist and bend deformations which are constant over the whole pitch

length. On the other hand, the N_{SB} phase has zero twist, but displays periodically alternating domains of splay and bend deformations. The N_{TSB} phase is intermediate between the N_{TB} and N_{SB} phase with properties of both, and is characterised by a uniform twist, and by periodic splay and bend deformations.

Supplementary Note 3: Typical configurations of the N_{TB}, Sm_{TSB}, and Sm_{SB} phases

In Supplementary Figure 4, Supplementary Figure 5, and Supplementary Figure 6 we show simulation configurations of the Sm_{SB}, Sm_{TSB}, and N_{TB} phases of hard curved spherocylinders with length $L/D = 19$ and opening angle $\Psi = 1.31$ along an expansion from packing fraction $\eta = 0.406$ to packing fraction $\eta = 0.367$, already shown in Figure 4 but complemented now with the top (xy) view and a schematic of the precession cone of the nematic director field.

Supplementary Note 4: Mapping of the phase diagram of thermotropic and lyotropic systems by matching the global nematic order parameter

In Figure 3 we compare the phase diagram of a thermotropic system of curved spherocylinders obtained from the Maier-Saupe theory with that of a lyotropic system of hard curved spherocylinders obtained from simulations. The Maier-Saupe theory describes the phase behaviour as a function of the (inverse) temperature $\beta\epsilon$, whereas the phase behaviour from simulations is described as a function of packing fraction η.

Following [2], we compare the thermotropic and lyotropic phase diagrams by mapping them as a function of the global nematic order parameter S_g. In particular, the global nematic order parameter S_g in a thermotropic nematic phase has the following power law behaviour

$$S_g(\beta\epsilon) = \left(1 - \alpha_{th}\left(\frac{\beta\epsilon_{IN}}{\beta\epsilon}\right)\right)^{\gamma_{th}}, \tag{2}$$

where $\beta\epsilon_{IN}$ is the bulk temperature at the isotropic-nematic (I-N) phase transition. Analogously, S_g in a lyotropic nematic phase follows the power law

$$S_g(\eta) = \left(1 - \alpha_{sim}\left(\frac{\eta_{IN}}{\eta}\right)\right)^{\gamma_{sim}}, \tag{3}$$

where η_{IN} is the packing fraction at the I-N phase transition.

To map the two phase diagrams, we proceed as follows. For each opening angle Ψ that we considered in theory (or simulations), we determine the inverse temperature $\beta\epsilon_{IN}(\Psi)$ (or packing fraction $\eta_{IN}(\Psi)$) at the bulk I-N/N_{TB} phase transition. We then measure the global nematic order parameter S_g as a function of $\beta\epsilon_{IN}(\Psi)/\beta\epsilon(\Psi)$ (or $\eta_{IN}(\Psi)/\eta(\Psi)$) for every opening angle Ψ. We then collect all the data for $S_g(\beta\epsilon_{IN}/\beta\epsilon)$ (or $S_g(\eta_{IN}/\eta)$) in Supplementary Figure 7 thereby ignoring the Ψ-dependence, and fit the data with Supplementary Equation 2 (or Supplementary Equation 3), which enables us to obtain an estimate of α_{th} and γ_{th} (or α_{sim} and γ_{sim}).

In Supplementary Figure 8 we compare the phase diagrams from theory and simulations as a function of the

global nematic order parameter S_g using Supplementary Equations 2 and 3 with $\alpha_\mathrm{th} = 0.98$, $\gamma_\mathrm{th} = 0.22$, $\alpha_\mathrm{sim} = 1.03$, $\gamma_\mathrm{sim} = 0.14$ obtained from the fits in Supplementary Figure 7, and with $\beta\epsilon_\mathrm{IN}(\Psi)$, and $\eta_\mathrm{IN}(\Psi)$, showing agreement between theory and simulations. We note that the isotropic state does not appear in this representation, as S_g of Supplementary Equations 2 and 3 is zero for $\beta\epsilon < \beta\epsilon_\mathrm{IN}$ and $\eta < \eta_\mathrm{IN}$, respectively.

Supplementary Note 5: Orientational order parameters, ellipticity, conical angle, and pitch from Maier-Saupe theory and simulations

In Supplementary Figure 9 we compare the global nematic order parameter S_g, smectic order parameter τ, ellipticity $e = \theta_\mathrm{b}/\theta_\mathrm{a}$, conical angle θ_a, and pitch length p for systems of curved spherocylinders for three representative opening angles Ψ as predicted by our Maier-Saupe theory as a function of inverse temperature $\beta\epsilon$ and as obtained from simulations as a function of packing fraction η. The inverse temperature in the Maier-Saupe theory plays a similar role as packing fraction in simulations, as thoroughly discussed in the main text and in the Supplementary Note 4. Theory and simulations are in good comparison. We clearly find that at low $\beta\epsilon$ (or η) the systems display an isotropic phase with $S_\mathrm{g} = 0$ and $\tau = 0$. Upon increasing $\beta\epsilon$ (or η), we find a transition to a uniaxial nematic N phase with $S_\mathrm{g} > 0$ and $\tau = 0$ for opening angle $\Psi = 0.71$ and 0.83 which transforms into an $\mathrm{N_{TB}}$ phase with $e > 0.8$, and $\theta_\mathrm{a} > 0$ at higher $\beta\epsilon$ (or η), whereas the isotropic phase transforms into an $\mathrm{N_{TB}}$ phase for $\Psi = 1.54$ (1.47) with $S_\mathrm{g} > 0$, $\tau = 0$, $e > 0.8$, and $\theta_\mathrm{a} > 0$. For even higher $\beta\epsilon$ (or η), the $\mathrm{N_{TB}}$ phase transforms into an $\mathrm{Sm_{TSB}}$ phase with a decreasing e and increasing τ upon increasing $\beta\epsilon$ (or η) until a transition occurs to $e < 0.2$ and $\theta_\mathrm{a} > 0$ corresponding to an $\mathrm{Sm_{SB}}$ phase. The only significant discrepancy lies in the fact that in simulations, the pitch length p and θ_a in the $\mathrm{Sm_{SB}}$ phase is always relatively small, whereas the Maier-Saupe theory shows an $\mathrm{Sm_{TSB}}$-$\mathrm{Sm_{SB}}$ phase transition with an abrupt change to the maximum possible conical angle $\theta_\mathrm{a} \simeq \pi/2$ and a relatively large pitch p along with $\theta_\mathrm{b} = 0$, and only at sufficiently high $\beta\epsilon$ a transition to an $\mathrm{Sm_{SB}}$ state with values of θ_a and p similar to the simulation results occurs (see for example Supplementary Figure 9e). We remark here that the absence of an $\mathrm{Sm_{SB}}$ phase with a conical angle $\theta_\mathrm{a} \simeq \pi/2$ and a relatively large pitch p may be caused by the finite size of the simulation box and the periodic boundary conditions. We find that compression of N and $\mathrm{N_{TB}}$ states in relatively small simulation boxes always result into an $\mathrm{Sm_{SB}}$ phase with a small pitch p and conical angle θ_a as shown in Supplementary Figure 10b, supposedly to preserve continuity in the pitch length and conical angle. However, if we perform simulations of a much larger system initialized in an $\mathrm{Sm_{SB}}$ phase with a conical angle $\theta_\mathrm{a} = \pi/2$, the system remains mechanically stable over a wide range of pressures as shown in Supplementary Figure 10a. Compression runs from an N or $\mathrm{N_{TB}}$ phase using larger simulation boxes are beyond the limits of our computational resources, and hence we cannot conclude which one of the two $\mathrm{Sm_{SB}}$ phases is more stable. However, the Maier-Saupe theory shows that the free energies of the two $\mathrm{Sm_{SB}}$ phases are very similar, but the $\mathrm{Sm_{SB}}$ phase with $\theta_\mathrm{a} \simeq \pi/2$ is slightly more stable. Finally, we remark that the smectic layer spacing $\lambda = p/2$ varies as $\lambda/L \in [0.32, 4.16]$, and perhaps the unidentified $\mathrm{Sm_X}$ phase with a smectic layer spacing of $\lambda \simeq 0.5L$ is actually an $\mathrm{Sm_{TSB}}$ or $\mathrm{Sm_{SB}}$ phase[3].

Supplementary Note 6: Equation of state in sedimentation and bulk

In Supplementary Figure 11, we present the equations of state of hard curved spherocylinders of aspect ratio $L/D = 19$ and opening angle $\Psi = 0.99$ as obtained from bulk simulations (pink line) and from integrating the density profile of a system subject to a gravitational field characterised with a gravitational length $l_{\mathrm{g}} = 7.5D$ (blue symbols). We find good agreement between the two equations of state.

Supplementary Note 7: Polar order and bend deformations

In Supplementary Figure 12, Supplementary Figure 13, and Supplementary Figure 14 we plot the x-, y-, and z-components of the polarization vector $\hat{\mathbf{m}}(z)$ measured for the exemplary $\mathrm{Sm_{SB}}$, $\mathrm{Sm_{TSB}}$, and $\mathrm{N_{TB}}$ phases of hard curved spherocylinders of aspect ratio $L/D = 19$ and opening angle $\Psi = 1.31$ reported in Figure 4 and in Supplementary Figures 4, 5, and 6. For comparison, we also plot the bend distortions of the nematic director as described by the bend vector $\hat{\mathbf{b}}(z) = \hat{\mathbf{n}} \times (\nabla \times \hat{\mathbf{n}}) \,/\, \|\hat{\mathbf{n}} \times (\nabla \times \hat{\mathbf{n}})\|$ calculated from the theoretical nematic director field $\hat{\mathbf{n}}_{\mathrm{TSB}}(z|\theta_{\mathrm{a}}, \theta_{\mathrm{b}}, q)$ in Equation 3, showing that the polarization vector $\hat{\mathbf{m}}(z)$ is always anti-parallel to the bend vector $\hat{\mathbf{b}}(z)$. Our results demonstrate that the formation of these spatially modulated phases is driven by spontaneous polar ordering coupled to bend deformations induced by the packing of curved particles.

For each exemplary state, we also report the x-, y-, and z-components of the measured nematic director field $\hat{\mathbf{n}}(z)$, the uniaxial order parameter $S(z)$, and the probability $\rho(z)$ of finding a particle at position z.

Finally, in Supplementary Figure 15 we show a fit of the nematic director field of the $\mathrm{Sm_{SB}}$ phase in Supplementary Figure 4 with our and Dozov's expressions for the nematic director field of the splay-bend nematic phase.

Supplementary References

1. Dozov, I. On the spontaneous symmetry breaking in the mesophases of achiral banana-shaped molecules. *EPL (Europhysics Letters)* **56,** 247 (2001).

2. Kwak, C. H. & Kim, G. Y. Graphical representations of the Maier–Saupe mean field theory in nematic liquid crystals. *Liquid Crystals* **46,** 1655–1665 (2019).

3. Mandle, R. J. & Goodby, J. W. Intercalated soft-crystalline mesophase exhibited by an unsymmetrical twist-bend nematogen. *CrystEngComm* **18,** 8794–8802 (2016).
